# Metataxonomic and metabolomic profiling revealed *Pinus koraiensis* cone essential oil reduced methane emission through affecting ruminal microbial interactions and host-microbial metabolism

**DOI:** 10.1186/s42523-024-00325-4

**Published:** 2024-06-28

**Authors:** Y. Choi, S. J. Lee, H. S. Kim, J. S. Eom, S. U. Jo, L. L. Guan, S. S. Lee

**Affiliations:** 1 Division of Applied Life Science (BK21), Gyeongsang National University, Jinju, 52828 Republic of Korea; 2 Institute of Agriculture and Life Science (IALS), Gyeongsang National University, Jinju, 52828 Republic of Korea; 3 Institute of Agriculture and Life Science and University-Centered Labs, Gyeongsang National University, Jinju, 52828 Republic of Korea; 4 Department of Agricultural, Food and Nutritional Science, University of Alberta, Edmonton, AB T6G 2P5 Canada; 5 Faculty of Land and Food Systems, The University of British Columbia, Vancouver, BC V6T 1Z4 Canada

**Keywords:** Essential oil, Enteric methane emission, Goat, Metataxonomics, Metabolomics

## Abstract

**Background:**

*Pinus koraiensis* cone essential oil (PEO) contains functional compounds such as monoterpene hydrocarbons, and the administration of PEO reduced methane (CH_4_) emissions during growing phase of goats. However, the mode of action of PEO driven CH_4_ reduction is not known, especially how the administration of PEO can affect rumen microbiota and host metabolism in goats during the fattening phase. This study aimed to elucidate the potential microbial and host responses PEO supplementation in goats using metataxonomics (prokaryotes and protozoa) and metabolomics (rumen fluid and serum).

**Results:**

Ten fattening Korean native goats were divided into two dietary groups: control (CON; basal diet without additives) and PEO (basal diet + 1.5 g/d of PEO) with a 2 × 2 crossover design and the treatment lasted for 11 weeks. Administration of PEO reduced CH_4_ concentrations in the exhaled gas from eructation by 12.0–13.6% (*P* < 0.05). Although the microbial composition of prokaryotes (bacteria and archaea) and protozoa in the rumen was not altered after PEO administration. MaAsLin2 analysis revealed that the abundance of *Selenomonas*, *Christensenellaceae* R-7 group, and *Anaerovibrio* were enriched in the rumen of PEO supplemented goats (*Q* < 0.1). Co-occurrence network analysis revealed that *Lachnospiraceae* AC2044 group and *Anaerovibrio* were the keystone taxa in the CON and PEO groups, respectively. Methane metabolism (*P* < 0.05) was enriched in the CON group, whereas metabolism of sulfur (*P* < 0.001) and propionate (*P* < 0.1) were enriched in the PEO group based on microbial predicted functions. After PEO administration, the abundance of 11 rumen and 4 serum metabolites increased, whereas that of 25 rumen and 14 serum metabolites decreased (*P* < 0.1). Random forest analysis identified eight ruminal metabolites that were altered after PEO administration, among which four were associated with propionate production, with predictive accuracy ranging from 0.75 to 0.88. Additionally, we found that serum sarcosine (serum metabolite) was positively correlated with CH_4_ emission parameters and abundance of *Methanobrevibacter* in the rumen (|*r*|≥ 0.5, *P* < 0.05).

**Conclusions:**

This study revealed that PEO administration reduced CH_4_ emission from of fattening goats with altered microbial interactions and metabolites in the rumen and host. Importantly, PEO administration affected utilizes various mechanisms such as formate, sulfur, methylated amines metabolism, and propionate production, collectively leading to CH_4_ reduction. The knowledge is important for future management strategies to maintain animal production and health while mitigate CH_4_ emission.

**Supplementary Information:**

The online version contains supplementary material available at 10.1186/s42523-024-00325-4.

## Background

Ruminants are unique in that they harbor a mutualistic microbial community consisting of prokaryotes and eukaryotes in their rumen that converts complex fibrous feed into valuable commodities, such as milk and meat for human’s consumption [1, 2]. Therefore, the rearing of ruminant livestock is crucial to address global food security challenges and reducing poverty, especially in a world with a rapidly growing population [3]. However, enteric CH_4_ emission, a natural process resulting from the rumen fermentation, from ruminant accounts for 30% of global anthropogenic CH_4_ emissions [4], significantly contributing to the global greenhouse gases emission. Moreover, the energy lost as CH_4_ from ruminants ranges between 2 and 12% of gross energy intake [5]. Therefore, it is important to find ways to mitigate CH_4_ emissions while maintaining production for the ruminant industry.

Numerous nutritional strategies have been used to reduce CH_4_ emissions in ruminants, enhance feed efficiency, and minimize energy loss from diets. Recently, nitrooxy compound (3-nitrooxypropanol, 3-NOP) and red seaweed (*Asparagopsis taxaformis*) have been found to be effective rumen CH_4_ inhibitors [6, 7] that can suppress rumen methanogenesis and improve production efficiency in long-term experiments [8, 9]. Although the mode of actions of these two mitigation methods have been established [10, 11], and the effect of 3-NOP on the composition of rumen microbes has been comprehensively studied [12, 13], only one study reported how 3-NOP affected function of rumen microbiome in dairy cows [14]. Additionally, Muizelaar et al. [15] reported that the bromoform from *A. taxiformis* could be excreted in urine and milk, causing abnormalities in the rumen papillae [14]. This raises concerns about the potential toxicity to rumen microbes and host ruminants. Therefore, it is necessary to identify new and natural sources that can reduce CH_4_ production without detrimental effects on ruminal fermentation and host, thereby providing an effective and sustainable strategy for controlling CH_4_ emissions.

Essential oils (EOs) are naturally derived from plants and contain many different chemical substances [16]. Some of these substances, such as monoterpene hydrocarbons (e.g., α-pinene, γ-terpinene, and D-limonene), are detrimental to rumen methanogens and protozoa, leading to reduced CH_4_ production [17, 18]. Furthermore, recent studies have reported that EOs can reduce rumen CH_4_ emissions in goats [19], dairy cows [20], and beef steers [21]. Previously, we reported that *Pinus koraiensis* cone essential oil (PEO) could reduce the CH_4_ production up to 65% in vitro [22] and the administration of PEO reduced CH_4_ concentrations by up to 16.5% in the exhaled gas from respiration in goats during the growing phase [19]. Additionally, the PEO supplementation decreased total VFA concentration, altered the composition of rumen bacteria, reduced the abundance of fungi and protozoa without affecting dry matter intake (DMI) of these goats suggesting that PEO has a potential to mitigate CH_4_ emissions and affect rumen microbial dynamics [19]. However, it decreased total VFA concentration and reduced the abundance of fungi and protozoa, while not impacting the abundance of methanogens. However, it is unclear if PEO affected microbial composition and metabolites in the rumen and host metabolism, and also if the PEO driven mitigate CH_4_ reduction is persistent in fattening goats.

We hypothesized that PEO administration leads to a shift in the microbial community and metabolic processes, contributing to decreased CH_4_ emissions while PEO does not affect host metabolism. In this study, we investigated the effects of PEO on CH_4_ emissions during the fattening phase in goats by examining its impact on the rumen microbial community (e.g., including bacteria and protozoa) as well as on rumen and serum metabolites. Such knowledge is important for future management strategies aimed at maintaining animal production and health while mitigating CH_4_.

## Results

### Growth performance, rumen fermentation parameters, and CH_4_ emissions

Administration of PEO did not affect the final body weight (BW), BW changes, DMI, or DM digestibility (DMD), pH and NH_3_-N concentrations in ruminal fluid compared to the CON group (Table 1). Methane concentrations in the gas exhaled from respiration (ppm-m, ppm/BW^0.75^, and ppm/kg DMI) did not differ between the CON and PEO groups (Table 2). However, when CH_4_ concentration was expressed as ppm/kg digestible DMI (DDMI), the CH_4_ concentration tended to be lower (*P* = 0.092) in the PEO group. Furthermore, eructation (ppm-m, ppm/BW^0.75^, and ppm/kg of DDMI) was significantly lower (*P* = 0.042, *P* = 0.044, and *P* = 0.036) in the PEO group compared to the CON group. Additionally, when CH_4_ concentration was expressed as ppm/kg DMI, the CH_4_ concentration during eructation tended to be lower (*P* = 0.079) in the PEO group than in the CON group. As a result, CH_4_ emissions from eructation in the PEO group were approximately 12%–13.6% compared to the CON group.

**Table 1:** DM intake, growth performance, and rumen fermentation characteristics for the dietary treatments

Item	Treatment		SEM	*P*-value
	CON	PEO		
Initial BW, kg	42.0	41.7	0.51	NS
Final BW, kg	41.2	41.2	0.55	NS
BW change^1^, kg	− 0.81	− 0.47	0.29	NS
DMI, kg/d	1.29	1.27	0.04	NS
DMD, %	0.70	0.70	0.02	NS
pH	7.09	7.00	0.07	NS
NH_3_-N, mg/dL	8.61	8.86	0.15	NS

NS: not significant; SEM: standard error of the mean; amplicon sequence variant; CON: without PEO; PEO: *Pinus koraiensis* essential oil; BW: body weight; DM: dry matter; DMI: dry matter intake; DMD: dry matter digestibility; NH_3_-N: ammonia nitrogen

^1^Over 28 d

**Table 2:** Enteric methane emission for the dietary treatments

Item	Treatment		SEM	*P* value
	CON	PEO		
CH_4_ from respiration				
ppm-m	16.9	16.0	0.75	NS
ppm/BW^0.75^	0.55	0.52	0.03	NS
ppm/kg of DMI	13.1	12.4	0.73	NS
ppm/kg of DDMI	24.4	22.6	0.90	< 0.1
CH_4_ from eructation				
ppm-m	61.2	53.6	3.15	< 0.05
ppm/BW^0.75^	1.98	1.74	0.10	< 0.05
ppm/kg of DMI	47.6	41.9	2.82	< 0.1
ppm/kg of DDMI	88.3	76.3	4.80	< 0.05

NS: not significant; SEM: standard error of the mean; CON: without PEO; PEO: *Pinus koraiensis* essential oil; CH_4_: methane; BW: body weight; DMI: dry matter intake; DDMI: digestible dry matter intake

### Alpha and beta diversity of rumen microbial communities did not alter after PEO administration

**Fig. 1:**
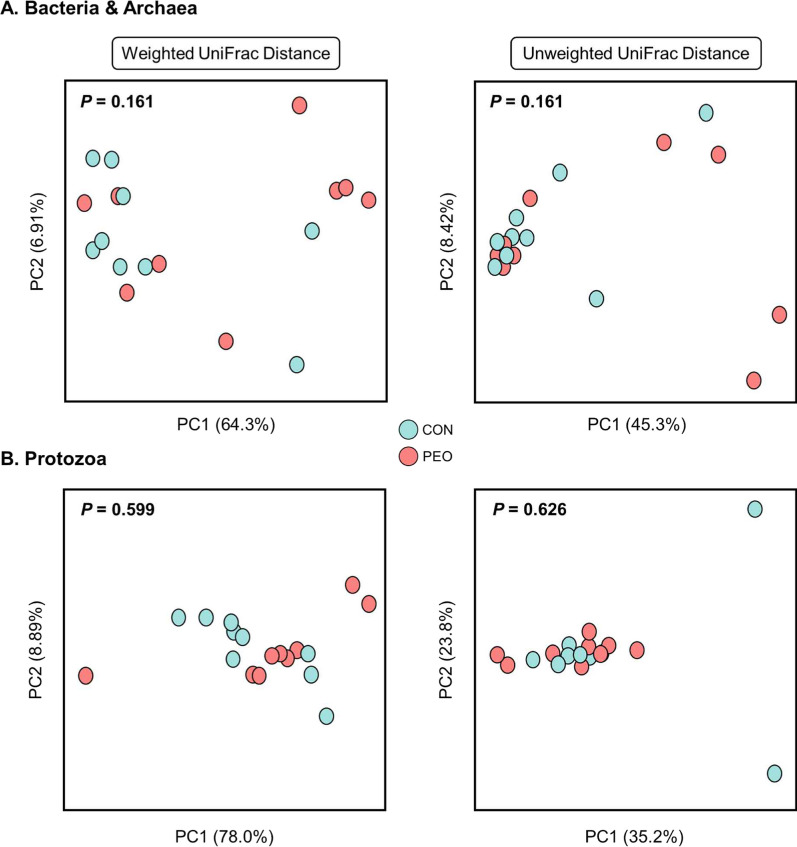
Principal coordinate analysis (PCoA) of the ruminal microbiota of **A** bacteria and archaea and **B** protozoa based on the matrices of Weighted UniFrac distance and Unweighted UniFrac distance. CON, without PEO; PEO, *Pinus koraiensis* cone essential oil

After denoising and quality filtration (Q-score > 20), an average of 73,306 ± 2842 (mean ± standard error) sequences were obtained for prokaryotes, with 51,079 ± 2062 sequences were retained for protozoa. Based on the minimum number of sequences (rarefaction curve) obtained for each kingdom across all samples, diversity analyses were performed using 54,511 and 34,579 sequences per sample for prokaryotes and protozoa, respectively. Good’s coverage index was 99.9% for all kingdom sequences, indicating that the sequencing depth adequately represented the rumen microbial community. The alpha diversity of each rumen kingdom (Chao 1 estimates, Evenness, Shannon's and Simpson's indices) had not significant differences between the CON and PEO groups (Table 3). Furthermore, PCoA and PERMANOVA revealed that none of the beta-diversity metrics, such as weighted and unweighted UniFrac distances, differed between the CON and PEO groups for each microbial kingdom in the rumen (Fig. 1).

**Table 3:** Summary of alpha diversity measurements of the rumen microbiota for the dietary treatments

Item	Treatment		SEM	*P* value
	CON	PEO		
Bacteria and archaea				
Chao1 estimate	628	533	82.1	NS
Evenness	0.78	0.76	0.02	NS
Shannon's index	7.18	6.75	0.37	NS
Simpson's index	0.98	0.97	0.01	NS
Protozoa				
Chao1 estimate	43	40	2.27	NS
Evenness	0.65	0.64	0.03	NS
Shannon's index	3.51	3.41	0.20	NS
Simpson's index	0.84	0.83	0.02	NS

NS: not significant; SEM: standard error of the mean; ASV: amplicon sequence variant; PD: phylogenetic diversity; CON**:** without PEO; PEO: *Pinus koraiensis* essential oil

### Some of the taxa of the rumen microbiota affected after PEO administration

In this study, only taxa with 0.1% of the average relative abundance present in at least 50% of the samples were used for downstream analysis.

**Fig. 2:**
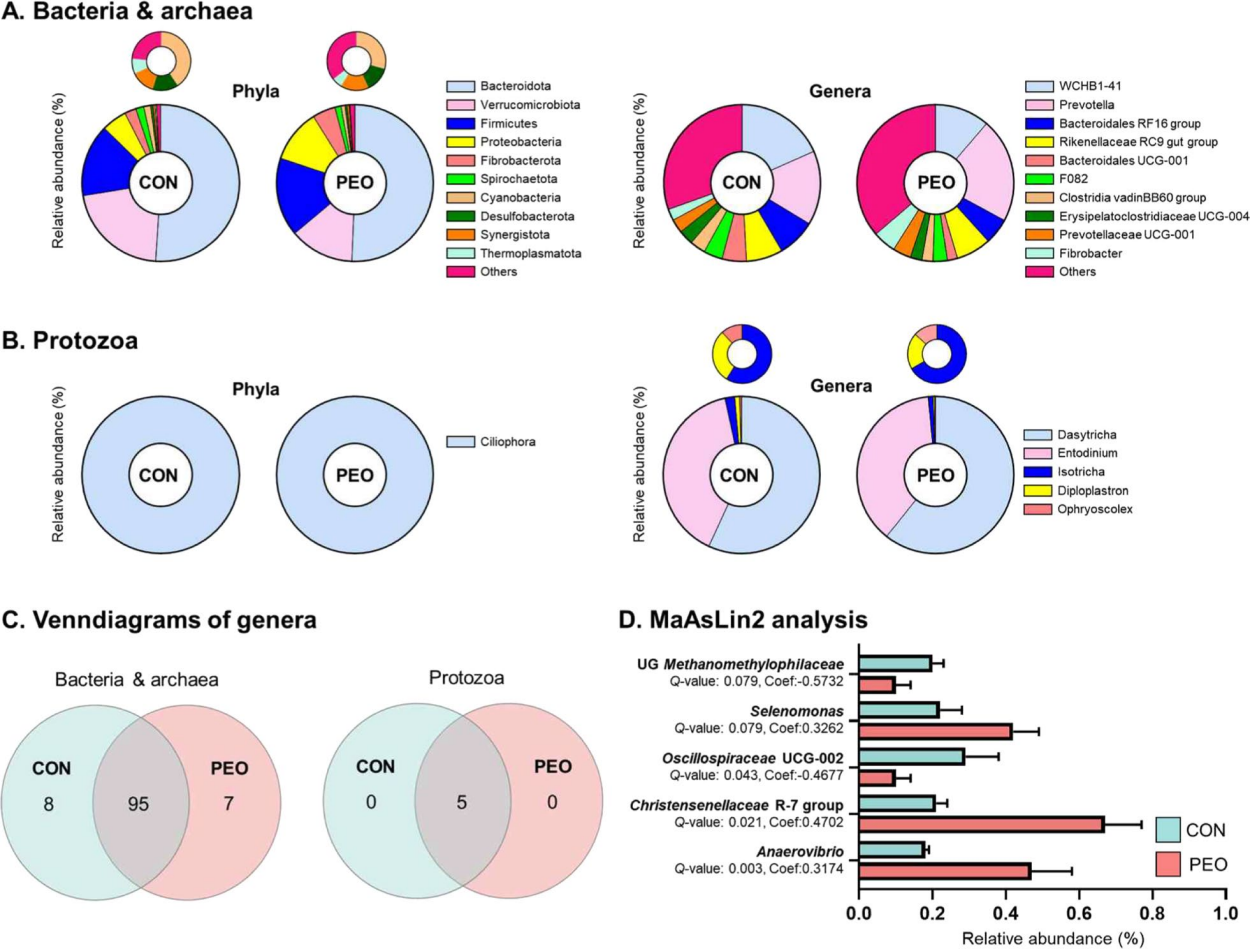
Compositional profiles of ruminal microbiota in goats, including **A** bacteria and archaea, **B** protozoa, and **C** venn diagrams showing the genera of rumen microbes shared between and unique to the CON and PEO group. **D** horizontal barplots showing the genera associated with the PEO group, compared to the CON group, as detected by MaAsLin2. Genera with Benjamini–Hochberg false discovery rate-adjusted *Q* < 0.1 were considered statistically significant for bacteria and archaea. Relative abundance of major phyla and genera (relative abundance ≥ 0.1% in more than 50% animals) for all individuals. CON, without PEO; PEO, *Pinus koraiensis* cone essential oil; Coef, coefficient; *Q*-value, *P* value corrected by the Benjamini–Hochberg method; MaAsLin2; microbiome multivariable association with linear models

For prokaryotes, 14 phyla, 38 families, and 58 genera of bacteria and 2 phyla, 2 families, and 2 genera of archaea were identified (Fig. 2A). For eukaryotes, 1 phylum, 1 family, and 5 genera of protozoa were identified (Fig. 2B). Among the prokaryotes, 95 of the 110 bacterial genera and two identified archaeal genera as well as all genera of protozoa were shared between the CON and PEO groups (Fig. 2C).

The dominant bacterial phyla in the rumen were Bacteroidota, Verrucomicrobiota, and Firmicutes, and two ruminal archaeal phyla were Thermoplasmatota and Euyarchaeota (Table S1). A total of 38 bacterial families were identified with *Prevotellaceae*, WCHB1-41, and *Rikenellaceae* being the most abundant (Table S2). In total, 58 bacterial genera were identified, the three most dominant genera being WCHB1-41, *Prevotella*, and *Bacteroidales* RF16 group (Table S3). MaASlin2 analysis identified differentially abundant microbial genera between the CON and PEO (Fig. 2D). UG *Methanomethylophilaceae* (*Q* = 0.079 and Coef = − 0.5732) and *Oscillospiraceae* UCG-002 (*Q* = 0.043 and Coef = − 0.4677) were enriched in the CON group compared to the PEO group, whereas *Selenomonas* (*Q* = 0.079 and Coef = 0.3262), *Christensenellaceae* R-7 group (*Q* = 0.021 and Coef = 0.4702), and *Anaerovibrio* (*Q* = 0.003 and Coef = 0.3174) were enriched in the PEO group. No significant difference was found for protozoa.

### Co-occurrence analysis revealed altered microbial interactions among different kingdoms in response to PEO administration

**Fig. 3:**
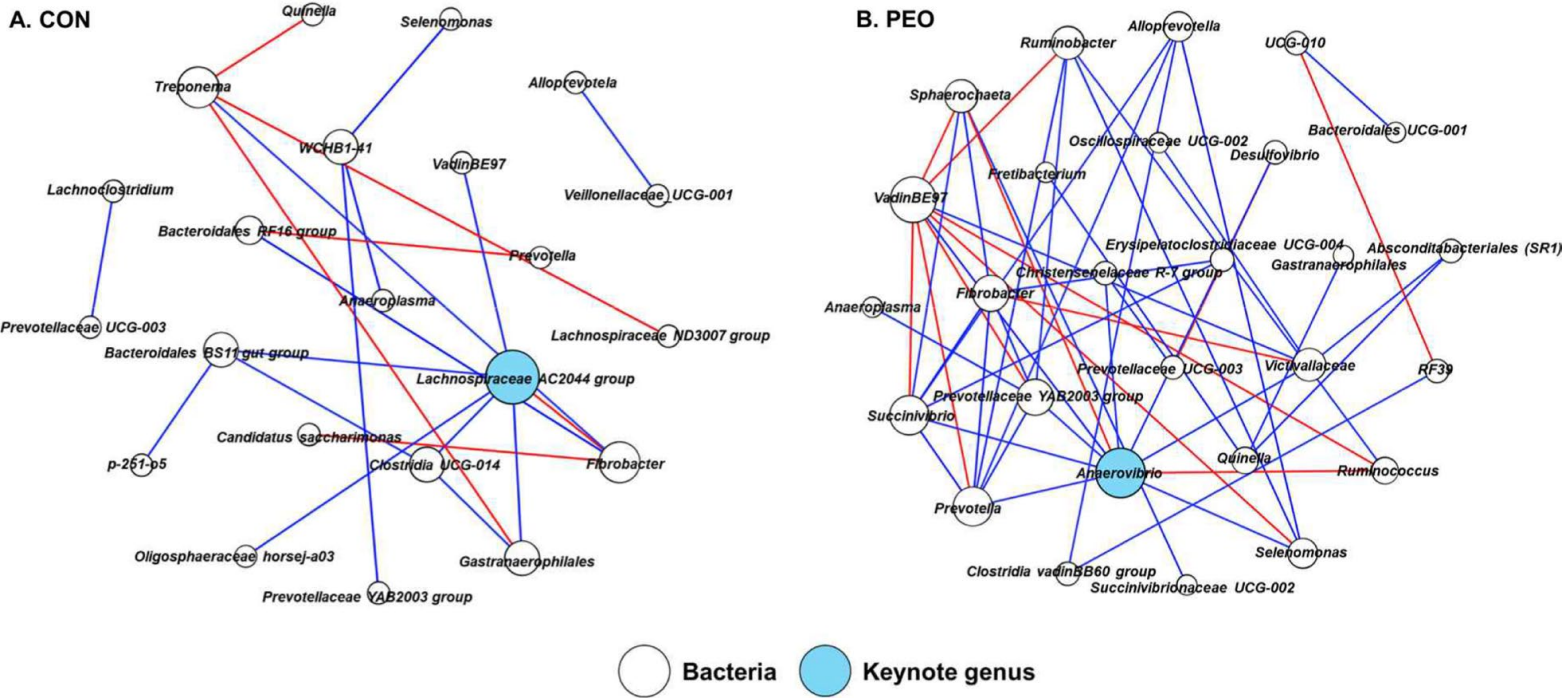
Exclusive co-occurrence and mutual exclusion microbial network in **A** CON and **B** PEO oral administration. The node color represents bacteria (white) and keystone genus (skyblue). The keystone genus is selected based on authority and eigenvector centrality measurements within each exclusive network. The edge color represents co-occurrence (blue) or mutual exclusive (red) interactions. The thickness of the edges is adjusted based on the absolute value of the correlation coefficients of each interaction. Only genera accounting for ≥ 0.1% average relative abundance in at least one of the treatments were used. CON, without PEO; PEO, *Pinus koraiensis* cone essential oil

Based on co-occurrence network analysis using CoDiNA [23], 21 and 48 significant interactions were identified among the 69 edges exclusively found in the ruminal microbiota of the CON and PEO groups, respectively (Fig. 3). While the CON group had 22 prokaryotic genus-level nodes, the PEO group had 27, with no protozoa or archaea found in either network (Table S4). In the CON group, *Lachnospiraceae* AC2044 group was identified as the keystone genus, co-occurring with five prokaryotic bacterial taxa (*Bacteroidales* BS11 gut group, *Clostridia UCG-014*, *Gastranaerophilales*, *Oligosphaeraceae horsej-a03*, and *VadinBE97*), while the *Fibrobacter* was mutually exclusive. In the PEO group, *Anaerovibrio* was identified as the keystone genus, co-occurring with eight prokaryotic bacterial taxa (*Christensenellaceae* R-7 group, *Prevotellaceae* YAB2003 group, *Prevotella*, *Selenomonas*, *Succinivibrio*, *Desulfovibrio*, *Victivallaceae*, and *VadinBE97*), while two prokaryotic bacterial taxa (*Ruminococcus* and *Sphaerochaeta*) were mutually exclusive.

### Predicted functional shifts of rumen bacteria and protozoa in response to PEO administration

**Fig. 4:**
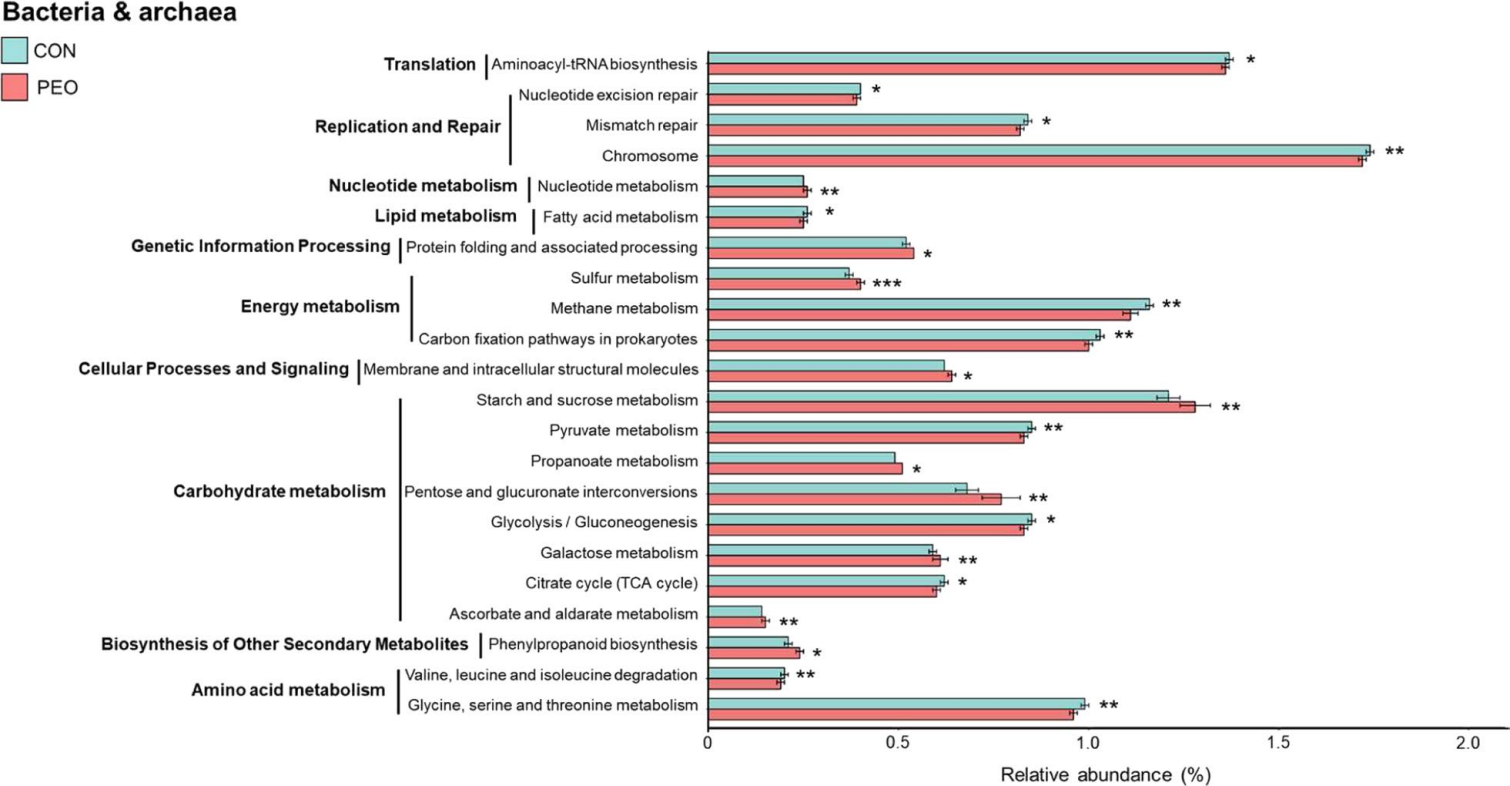
Predicted prokaryotic functions (CowPI database) detected using LEfSe (LDA > 2.0, *P* < 0.05) in the ruminal microbiota of CON and PEO groups. Only the functional parameters accounting for ≥ 0.1% average relative abundance in at least one of the treatments were statistically analyzed by LEfSe. CON, without PEO; PEO, *Pinus koraiensis* cone essential oil; LDA: linear discriminant analysis; LEfSe: linear discriminant analysis effect size. *P < 0.1, **P < 0.05, ***P < 0.001

Based on the predicted functional characteristics of rumen bacteria using CowPI [24], a specific functional inference tool for the rumen microbiome, carbohydrate metabolism was tentatively higher (*P* = 0.063) in the PEO group compared to the CON group. The PEO group exhibited enrichment of functions in ascorbate and aldarate metabolism (*P* = 0.022), galactose metabolism (*P* = 0.023), pentose and glucuronate interconversions (*P* = 0.008), and starch and sucrose metabolism (*P* = 0.019), whereas the CON group showed enrichment of functions in the citrate cycle (TCA cycle) (*P* = 0.069), glycolysis/gluconeogenesis (*P* = 0.078), propionate metabolism (*P* = 0.059), and pyruvate metabolism (*P* = 0.017) (Fig. 4). Compared to the PEO group, the CON group exhibited enriched functions in two amino acid metabolism pathways and one lipid metabolism pathway. Furthermore, CH_4_ metabolism (*P* = 0.010) and carbon fixation pathways in prokaryotes (*P* = 0.034) were enriched in the CON group, whereas sulfur metabolism (*P* = 0.003) was enriched in the PEO group. No functions were predicted for the protozoal community due to the absence of an appropriate database.

**Fig. 5:**
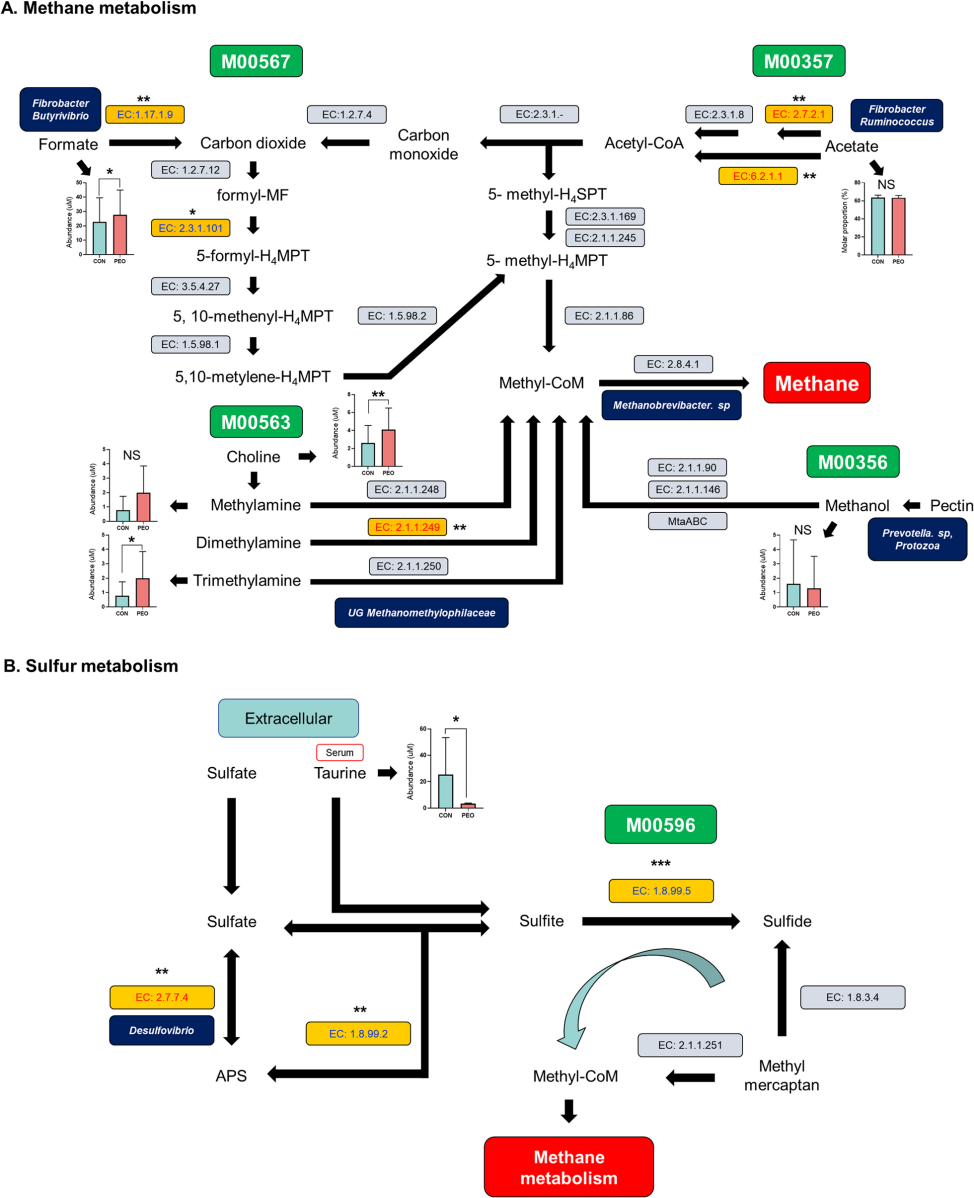
Differential abundance of enzymes involved in **A** methane and **B** sulfur metabolism in the CON and PEO groups. Enzymes involved in these metabolic modules are shown in yellow. The blue text represents enzymes enriched in the PEO group, while the red text indicates enzymes enriched or tending to be enriched in the CON group. Inside the navy rectangles are the rumen microbiota that play an important role in the pathway. *P < 0.1, **P < 0.05, ***P < 0.01. Metabolic modules include: M00567: methanogenesis, carbon dioxide to methane, M00563: methanogenesis, methylamine/dimethylamine/trimethylamine to methane, M00357: methanogenesis, acetate to methane, M00356: methanogenesis, methanol to methane, M00596: dissimilatory sulfate reduction, sulfate to hydrogen sulfide (H_2_S)

Differential abundance of enzymes involved in the four predominant ruminal methanogenesis modules [M00567: methanogenesis, carbon dioxide (CO_2_) to CH_4_; M00563: methanogenesis, methylamine/dimethylamine/trimethylamine to CH_4_; M00357: methanogenesis, acetate to CH_4_; M00356: methanogenesis, methanol to CH_4_] in CON and PEO groups were predicted (Fig. 5A). No significant differences were observed in the individual methanogenesis modules. In M00567, EC 1.17.1.9 (*P* = 0.015) significantly enriched, while EC 2.3.1.101 (*P* = 0.092) was tended to be enriched in the PEO group. In M00563, EC 2.1.1.249 (*P* = 0.050) significantly enriched in the CON group. Similarly, both EC 2.7.2.1 (*P* = 0.002) and EC 6.2.1.1 (*P* = 0.009) belong to M00563, were also found to be significantly enriched in the CON group. No significant differences were observed in M00356. Additionally, [M00596: dissimilatory sulfate reduction, sulfate to hydrogen sulfide (H_2_S)], which constitutes one of the sulfur metabolism and hydrogen (H_2_) sink pathways, showed significant enrichment (*P* = 0.007) in the PEO group (Fig. 5B). In M00596, EC 2.7.7.4 (*P* = 0.019), EC 1.8.99.2 (*P* = 0.022), EC 1.8.99.5 (*P* = 0.007) significantly enriched in CON and PEO groups, respectively. Furthermore, the genera of the related enzymes are also summarized in Fig. 5A and B.

### Rumen and serum metabolome analyses revealed microbial and host metabolites affected by PEO administration

We identified 181 metabolites in rumen fluid and 162 in serum that were present in at least 50% of the samples. Of these, 36 metabolites in rumen fluid (Table S5) and 18 in serum (Table S6) showed significant changes after PEO administration (*P* < 0.1). In particular, 11 rumen metabolites had higher abundances in the PEO group, while 25 had lower abundances. In the serum, 4 metabolites had higher abundances in the PEO group, while 14 had lower abundances.

**Fig. 6:**
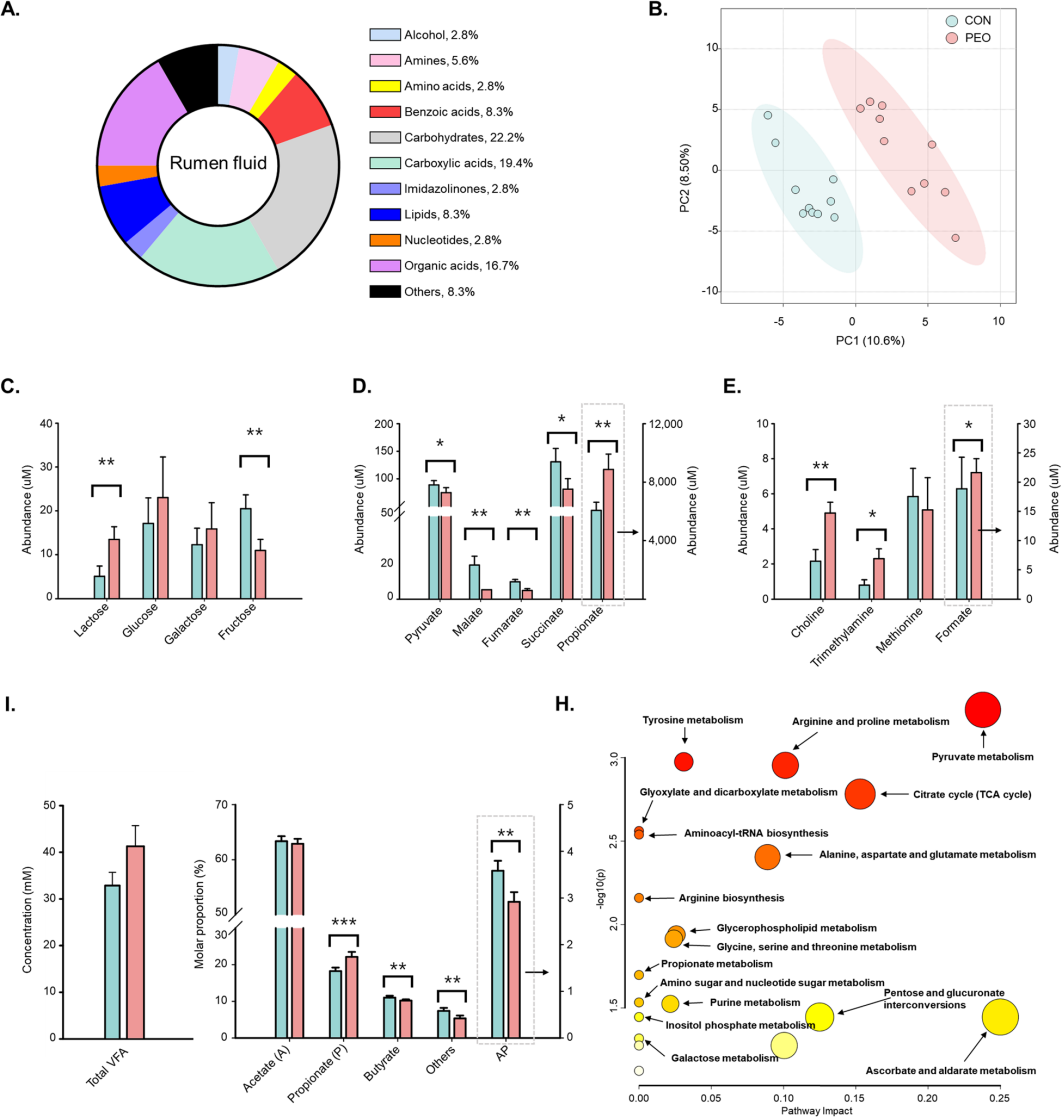
**A** Classification of measured metabolites according to chemical class in rumen fluid. **B** partial least square discriminant analysis (PLS-DA) score plot of rumen fluid. **C** abundance of carbohydrates, **D** abundance of propionate precursors and propionate, **E** abundance of choline, trimethylamine, and formate, **I** concentration of total VFA, molar proportions of individual VFAs, and AP ratio. **H** metabolic pathway mapping of common quantified metabolites in the rumen fluid. Selected metabolites met the criteria of *P* < 0.1 and VIP score ≥ 1.5. CON, without PEO; PEO, *Pinus koraiensis* cone essential oil; VFA, volatile fatty acid; Others, sum of valerate, isovalerate and isobutyrate; AP, acetate to propionate; VIP, variable importance in projection. *P < 0.1, **P < 0.05

For rumen fluid metabolites, they were classified into 11 groups (Fig. 6A). The PLS-DA score plots were clearly separated from the total variation between the CON and PEO groups (Fig. 6B). After PEO administration, differences in abundances were observed carbohydrates (lactose, glucose, galactose, and fructose; Fig. 6C), propionate precursors (pyruvate, malate, fumarate, succinate, and propionate; Fig. 6D), and other metabolic intermediates related to CH_4_ and sulfur metabolism (choline, trimethylamine, methionine, and formate; Fig. 6E). In the PEO group, lactose, propionate, choline, trimethylamine, and formate were higher compared to the CON group, whereas fructose, pyruvate, malate, fumarate, and succinate were lower. The total VFA and molar proportions of individual VFAs are shown in Fig. 6F. There were 17 metabolic pathways had significant changes after PEO administration with false discovery rate (FDR) < 0.1 (Fig. 6G). Detailed information on *P* and FDR values of rumen fluid metabolites and pathways are shown in Tables S5 and S7.

**Fig. 7:**
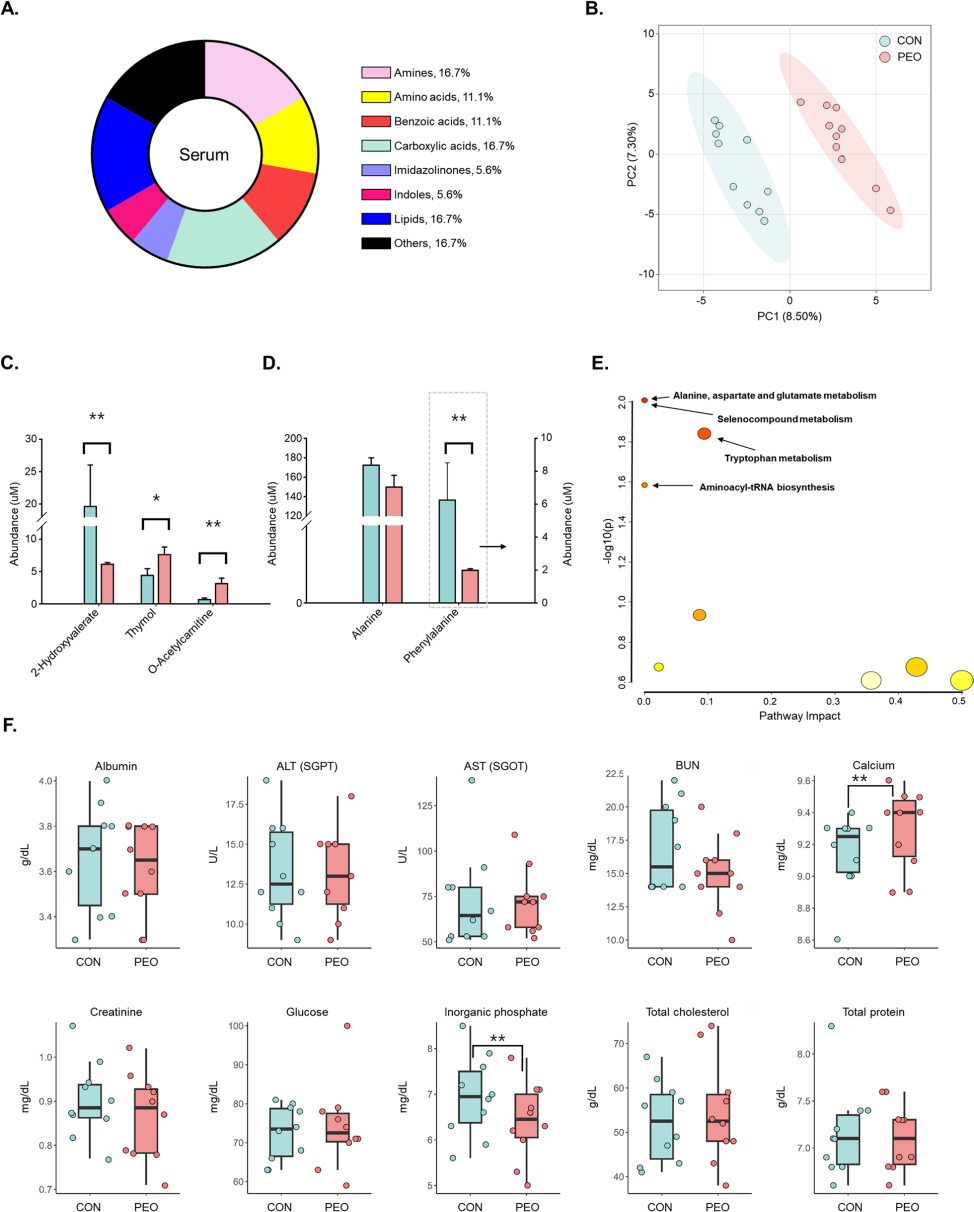
**A** Classification of measured metabolites according to chemical class in serum using ^1^H-NMR. **B** partial least square discriminant analysis (PLS-DA) score plot of serum. **C** abundance of lipids and **D** amino acids. **E** metabolic pathway mapping of common quantified metabolites in the serum. **F** abundance of serum metabolites and liver enzymes using UV spectroscopy and colorimetry method. Selected metabolites obtained from ^1^H-NMR met the criteria of *P* < 0.1 and VIP score ≥ 1.5. CON, without PEO; PEO, *Pinus koraiensis* cone essential oil; ALT/SGPT, alanine transaminase/serum glutamic pyruvate transaminase; AST/SGOT, aspartate aminotransferase/serum glutamic oxaloacetic transaminase; BUN: blood urea nitrogen. *P < 0.1, **P < 0.05

Serum metabolites were classified into eight groups (Fig. 7A). The PLS-DA score plots were clearly separated from the total variation between the CON and PEO groups (Fig. 7B). After PEO administration, differences in abundances were observed in lipids (2-hydroxyvalerate, thymol, and O-acetylcarnitine; Fig. 7C) and amino acids (alanine and phenylalanine; Fig. 7D). In the PEO group, thymol and O-acetylcarnitine were higher, whereas 2-hydroxyvalerate, alanine, and phenylalanine were lower compared to the CON group. Based on metabolites that showed significant changes with PEO administration, four metabolic pathways were identified with an FDR < 0.1 (Fig. 7E). Additionally, we also measured serum contents such as albumin, alanine transaminase/serum glutamic pyruvate transaminase (ALT/SGPT), aspartate aminotransferase/serum glutamic oxaloacetic transaminase (AST/SGOT), blood urea nitrogen (BUN), calcium, creatinine, glucose, inorganic phosphate, total cholesterol, and total protein (Fig. 7F). Of these, only two parameters were significantly affected. For example, calcium levels were significantly higher (*P* = 0.042) in the PEO group than in the CON group, whereas inorganic phosphate levels were significantly higher (*P* = 0.048) in the CON group than in the PEO group. Detailed information on *P* and FDR values of rumen fluid metabolites and pathways are shown in Tables S6 and S8.

### Correlation analysis among differential rumen microbial taxa, rumen and serum metabolites, and animal performance

**Fig. 8:**
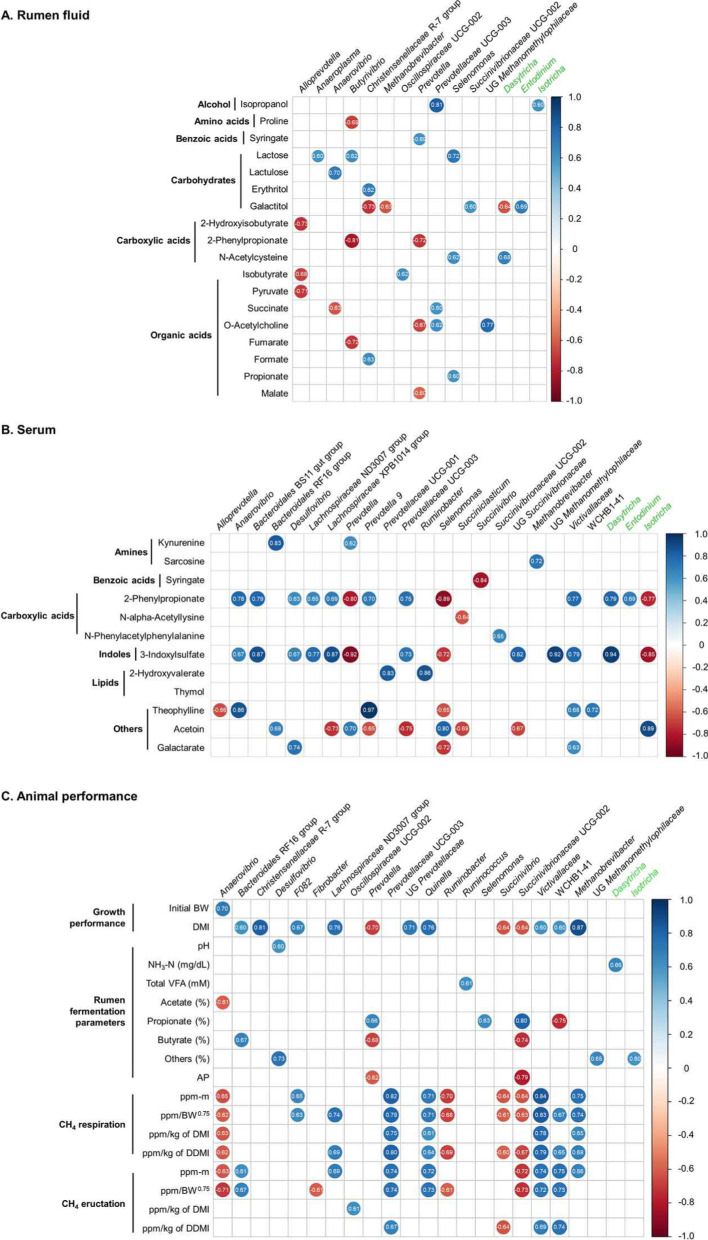
Correlation of **A** rumen and **B** serum metabolites, and **C** animal performance parameters with the relative abundance of major bacterial and archaeal and protozoal (green) genera (occupying over 0.1% average relative abundance in at least one of the treatments). Correlation analyses were conducted using Spearman’s rank correlation. Only strong correlation coefficients (|*r*|≥ 0.6) and significant (*P* < 0.05) correlations were selected to be shown on the plot. BW, body weight; CH_4_, methane; DMI, dry matter intake; DDMI, digestible dry matter intake; NH_3_-N, ammonia nitrogen; Others, valerate, isovalerate, and isobutyrate; AP, acetate to propionate

Significant and strong Spearman's rank correlations (|*r*|≥ 0.6, *P* < 0.05) were found between some of the rumen and serum metabolites and several ruminal major microbial genera (Fig. 8). In the rumen fluid (Fig. 8A), *Selenomonas* was positively correlated with ruminal lactose (*r* = 0.722, *P* = 0.002), N-acetylcysteine (*r* = 0.616, *P* = 0.011), and propionate (*r* = 0.600, *P* = 0.014). Moreover, *Anaerovibrio* was positively correlated with ruminal lactulose (*r* = 0.701, *P* = 0.003) and negatively correlated with succinate (*r* = − 0.629, *P* = 0.009) abundance. The *Christensenellaceae* R-7 group was positively correlated with ruminal erythritol (*r* = 0.689, *P* = 0.003) and formate (*r* = 0.632, *P* = 0.009) but negatively correlated with galactitol (*r* = − 0.733, *P* = 0.001).

Furthermore, *Anaerovibrio* was positively correlated with serum 2-phenylpropionate (*r* = 0.784, *P* = 0.007) and 3-indoxylsulfate (*r* = 0.667, *P* = 0.035) (Fig. 8B). *Selenomonas* was negatively correlated with serum 2-phenylpropionate (*r* = − 0.889, *P* = 0.001), 3-indoxylsulfate (*r* = − 0.722, *P* = 0.018), theophylline (*r* = − 0.646, *P* = 0.044), and galactarate (*r* = − 0.721, *P* = 0.009) and positively correlated with acetoin (*r* = − 0.629, *P* = 0.009). *Methanobrevibacter* and UG *Methanomethylophilaceae* were positively correlated with serum sacrcosine (*r* = 0.721, *P* = 0.019) and 3-indoxylsulfate (*r* = 0.920, *P* < 0.001), respectively.

Regarding animal performance, only *Selenomonas* was positively correlated with the molar proportion of propionate (*r* = 0.628, *P* = 0.009) (Fig. 8C). DMI was positively correlated (|*r*|≥ 0.6, *P* < 0.05) with *Christensenellaceae* R-7 group and *Methanobrevibacter*, whereas it was negatively correlated *Prevotella*, *Succinivibrio*, and *Succinivibrionaceae* UCG-002 (|*r*|≥ 0.6, *P* < 0.05). Furthermore, protozoal genera, such as *Dasytricha* and *Isotricha*, were positively correlated with NH_3_-N (*r* = 0.657, *P* < 0.001) and the molar proportion of other VFAs (sum of valerate, isovalerate and isobutyrate) (*r* = 0.601, *P* = 0.001), respectively. Most of the CH_4_ emission parameters were negatively correlated (|*r*|≥ 0.6, *P* < 0.05) with *Anaerovibrio*, *Succinivibrio*, and *Succinivibrionaceae* UCG-002, whereas positively correlated (|*r*|≥ 0.6, *P* < 0.05) with *Prevotellaceae* UCG-003, *Quinella*, *Victivallaceae*, WCHB1-41, and *Methanobrevibacter*.

### Microbe–metabolite interaction patterns associated after PEO administration

**Fig. 9:**
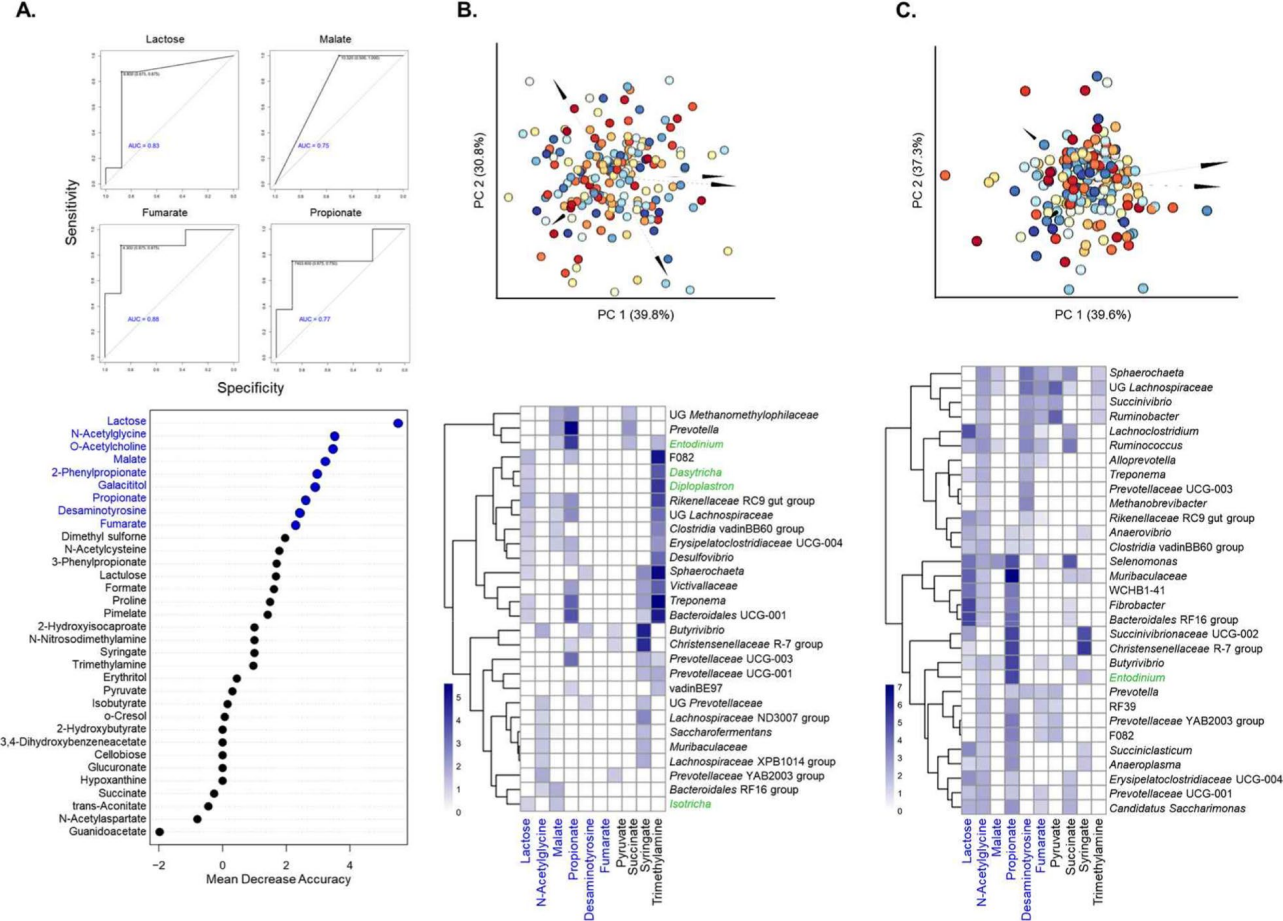
Prediction of microbe and metabolite co-occurrences in Korean native goats between CON and PEO group. **A** receiver operating characteristic (ROC) curve and confusion matrix for the random forest model using the eight selected metabolites (shown in navy) with mean decrease accuracy > 2. Biplot drawn from the microbe‑ metabolite vectors (mmvec) co-occurrence probabilities estimated for the dataset of **B** CON and **C** PEO groups. Axes correspond to principal components from the singular value decomposition of the microbe-metabolite co-occurrence probabilities estimated using mmvec. Microbes are represented by arrows and metabolites by dots. Heatmaps display the inferred co-occurrence probabilities for various metabolites given the presence of specific microbial taxa in the rumen of goats under **B** CON and **C** PEO groups. Colors indicate genera of bacteria (black) and protozoa (green)

After employed a neural network-based approach using microbe-metabolite vectors (mmvec), a method capable of predicting metabolite abundance profiles from individual microbial sequences [25], we identified 33 rumen metabolites significantly altered after PEO administration (*P* < 0.05). Further, random forest (RF) model was applied to predict the CH_4_ phenotypes using rumen metabolites and we found eight selected metabolites *N*-acetylglycine, *O*-acetylcholine, malate, 2-phenylpropionate, galactol, propionate, desaminotyrosine, and fumarate; each with a mean decrease in accuracy (MDA) greater than 2 (Fig. 9A). The MDA scores, which indicate the importance of each metabolite within the model, were calculated based on the increase in prediction error when each metabolite was removed from the training dataset predictors. Particularly, our model demonstrated high predictive accuracy (AUC between 0.75 and 0.88) four of these metabolites (lactose, malate, fumarate, and propionate) as related to propionate production, indicating their potential link to CH_4_ emission reduction. Based on these results, heat maps were generated to visualize the inferred co-occurrence probabilities (> 2) of specific metabolites (MDA > 2), revealing distinct interaction patterns between rumen microbes and metabolites across both groups (Fig. 9B and C).

## Discussion

Although our study used laser methane detector (LMD) to assess CH_4_ emission that did not allow the individual data collection, many studies have reported using the LMD to measure CH_4_ emissions from dairy cows [26, 27], beef steers [28, 29], and goats [19, 30, 31] due to its advantages include being cost-effective, flexible, and portable. Recently, a detailed summary and discussion about the its advantages and limitations of LMD by Sorg et al. [32], highlighted the limitation in CH_4_ measurement accuracy is only moderate, as it measures concentrations rather than quantities, and it is strongly affected by environmental conditions. To overcome these limitations, we used the automatic multi-scale peak detection (AMPD) algorithm and a double normal distribution for data processing to detect and separate CH_4_ concentration peaks into respiration and eructation, assuming the mean of the normal distribution as the representative point measurement of CH_4_ concentration for each event [29], suggesting that this process has led to more accurate CH_4_ measurement by LMD in this study.

Administration of PEO reduced CH_4_ emission, without significantly impacting growth performance of goats during the fattening phase, confirmed the previous findings in growing goats [19]. This suggests that the PEO driven CH_4_ reduction is persistent through the key production stages of goats. Although both experiments showed that DMI and BW of goats were not affected by PEO administration, we observed a noticeable alteration in the proportions of individual VFAs [e.g., propionate, butyrate, and other VFAs (sum of valerate, isovalerate and isobutyrate)]. Our previous studies found that the administration of PEO lowered total VFA concentration and NH_3_-N in the rumen during the growing phases of goats [19]. However, these effects were not observed during the fattening stage. There was an increase in the proportion of propionate and a decrease in butyrate in the rumen of PEO supplemented finishing goats (this study), while the proportion of propionate decreased, and butyrate increased in the growing goats compared to the CON group [19]. Both butyrate and propionate are crucial in reducing CH_4_ emissions, as their production in the rumen competes with methanogenesis for metabolic H_2_, thereby potentially reducing CH_4_ production [18]. In the growing phase, the PEO group exhibited a higher abundance of butyrate-producing bacteria such as *Oscillospira*, which may lower CH_4_ emissions by reallocating electrons from CH_4_ production to butyrate synthesis [33]. Conversely, during the fattening phase, the observed increase in propionate is likely due to a higher presence of propionate-producing bacteria such as *Anaerovibrio*, *Succinivibrio*, and *Succinivibrionaceae* UCG-002, further supporting the reduction of CH_4_ emissions by competing with methanogenesis for metabolic H_2_ in the rumen [34]. It is noticeable that the same feed types were used for two studies with only increased amount of feed for goats during the fattening phase. These suggest the observed CH_4_ changes could be the results of administration of PEO which influence the metabolic pathways and microbial compositions responsible for VFA and CH_4_ production of the rumen in goats during the growing or fattening phase.

Although the diversity indices of rumen microbial community were not affected by the administration of PEO, some ruminal bacteria and archaea were differentially abundant between the CON and PEO groups. For example, the relative abundance of succinate-producing bacteria such as *Succinivibrio*, *Succinivibrionaceae* UCG-002, and *Ruminobacter* were higher in the PEO group. Members of *Succinivibrionaceae* have been reported to be negatively correlated with the abundance of methanogens [35] and positively associated with higher feed efficiency [36] and lower CH_4_ production [37], suggesting that PEO could promote the growth of these bacterial taxa, leading to higher propionate production and reduced CH_4_ concentrations in the exhaled gas from eructation. Furthermore, CH_4_ emission parameters exhibited negative correlation with succinate and propionate producing bacteria, such as *Anaerovibrio*, *Succinivibrio*, and *Succinivibrionaceae* UCG-002, aligning with enhanced propionate metabolism and enriched these three genera in the PEO group. Additionally, PEO administration led to an increase in the abundance of *Anaerovibrio*, *Prevotella*, and *Selenomonas*, which produce propionate and consume H_2_ via the succinate pathway during the fermentation of sugars and lactate [27]. This process is likely pivotal for H_2_ utilization, indicating a significant shift where H_2_ is predominantly used for propionate production rather than being diverted to CH_4_ production. Indeed, propionate precursors (e.g., pyruvate, malate, fumarate, and succinate) showed lower abundances in the PEO group, suggesting that microbes enriched by PEO administration play a significant role in efficiently converting these precursors into propionate.

Contrary to expectations, our results indicated a high enrichment of enzymes associated with the CO_2_ to CH_4_ pathway, such as EC 1.17.1.9 and EC 2.3.1.101, in the rumen of PEO group, suggesting a potential acceleration in CH_4_ production. However, CH_4_ emissions were reduced in the PEO group. This contradictory result may be attributed to a lack of enrichment or reduced activity of downstream enzymes necessary for the final steps of CH_4_ production. Alternatively, it could be due to a lower involvement of key microbial populations that contribute to CH_4_ emission. Regardless, our findings suggest that PEO administration could alter microbial community dynamics and inhibit certain stages of the methanogenesis pathway.

This study further identified rumen metabolite–microbe relationships. Diverse rumen microbiota, including bacteria, protozoa, and fungal genera, were associated with trimethylamines (Fig. 9B and C), suggesting that these microbes have a potential synergistic effect to affect the metabolism of these metabolites. Notably, *Desulfovibrio* possesses the (cutC) gene, which may degrade choline to trimethylamine [38]. The mmvec results revealed a relatiomship between *Desulfovibrio* and trimethylamine, as well as with UG *Methanomethylophilaceae* in the CON group (Fig. 9B). UG *Methanomethylophilaceae*, a family within *Methanomassiliicoccales* [39] known for utilizing methylated amines (methyl-, dimethyl-, and trimethylamine) to produce CH_4_ [40], showed a positive correlation with *Desulfovibrio* (*r* = 0.793, *P* < 0.001) and CH_4_ metabolism (*r* = 0.613, *P* = 0.004) (Fig. S1) in this study. Moreover, EC 2.1.1.249 was enriched in the CON group, an enzyme known to degrade dimethylamine to produce CH_4_ [41], however the dimethylamine abundance was not detected. The PEO group showed higher abundance of choline and methylated amines than the CON group, suggesting that the lower abundance of UG *Methanomethylophilaceae* may have limited capacity for utilizing these methylated amines, leading to their accumulation. According to Zhou et al. [42], accumulated amines in the rumen can be absorbed into the blood and transformed into trimethylamine N-oxide in the liver. However, no significant difference was observed in serum trimethylamine N-oxide abundance between the two groups (CON: 6.90 ± 1.15 vs. PEO: 7.45 ± 0.82, *P* = 0.657). One possible explanation for the lack of significant difference in serum trimethylamine N-oxide abundance is its excretion in urine [43]. Taken together, these findings suggest that *Desulfovibrio* may be responsible for degrading choline to trimethylamine, and UG *Methanomethylophilaceae* may have more opportunities to utilize trimethylamine, thereby resulting in increased CH_4_ production.

Additionally, using random forest analysis, we identified eight ruminal metabolites that were predictive for reduced CH_4_ after PEO administration with predictive accuracy ranging from 0.75 to 0.88. Among them, four metabolites (e.g., lactose, malate, fumarate, and propionate) are major representatives of propionate metabolism [44]. Succinate- and propionate-producing bacteria (e.g., *Selenomonas*, *Succinivibrionaceae* UCG-002, *Succiniclasticum*, and *Anaerovibrio*) exhibited strong co-occurrence. This is consistent with previous studies on dairy cattle, where *Selenomonas* co-occurred with the family *Succinivibrionaceae* [45]. Given their ecological functions and the enrichment of propionate metabolism in both rumen microbiota and metabolites in the PEO group, it is plausible that they exhibit positive interactions and serve as pivotal bacteria in the rumen.

Our study further revealed enriched sulfur metabolism in the PEO group. Administering sulfate or elemental sulfur effectively reduces ruminal CH_4_ emissions by diverting ruminal H_2_ away from CH_4_ production in goats [46], suggesting potential mechanisms of PEO affecting microbial sulfur metabolism for reducing CH_4_ emissions [47]. Sulfur-reducing bacteria including *Desulfovibrio*, *Desulfohalobium*, and *Sulfolobus*, may accelerate sulfur metabolism and compete with methanogens for H_2_ in the rumen [48, 49]. Although the abundance of *Desulfovibrio* did not significantly differ between the CON and PEO groups, an enrichment in sulfate reduction function (EC 1.8.99.5) in the PEO group suggests a potentially limiting H_2_ availability and inhibiting its contribution to CH_4_ metabolism (Fig. 5B). Moreover, we found sulfur-containing amine taurine in the serum was lowered in the PEO group. Taurine is known to function as an anaerobic electron acceptor [50], a recent study reported taurine supplementation reduced CH_4_ production in vitro [51]. During sulfur metabolism, taurine can degrade into sulfide, which serving as an alternative H_2_ sink [47]. Therefore, it is possible to hypothesize that the decreased taurine abundance after PEO administration contributes to electron consumption, thereby enhancing sulfur metabolism and ultimately reducing the electrons available for CH_4_ production which warrants to be further studied.

In addition to the observed microbial taxa, metabolites and predicted functional difference, we found the PEO could affect the interactions among different microbial groups and key hub microbes. Notably, the ruminal microbiota of the PEO group exhibited more interactions (co-occurrence and mutual-exclusion) compared to the CON group (27 vs. 22). *Anaerovibrio* was denoted as the keystone taxon in the PEO group, which produces both succinate and propionate [52]. This genus was co-occurrence with propionate producing bacteria such as *Selenomonas*, *Succinivbrio*, and *Prevotella*. Archaea and protozoa were not identified in the networks of both CON and PEO groups. This could be attributed to exclusive nodes, which represent 50% and 59% of the overall ruminal microbial communities, indicating that differences in rumen fermentation and animal phenotypes may arise from shared or undefined microbial networks that occurred in each group (Table S4).

This study also identified the altered rumen taxa and how they can contribute to the metabolite compositions in both the rumen and the serum. The observed metabolites may have positive and/or negative effects on goats’ metabolism. Recently, Yanibada et al. [53] have reported an association between high serum abundances of kynurenine and serotonin and CH_4_ inhibition. Based on our result, serum kynurenine abundance was higher in the PEO group, whereas serotonin was lower. Despite these observations, no correlation was found between CH_4_ emission parameters and these metabolites. Instead, we found that sarcosine, derived from choline, was positively correlated with CH_4_ emission parameters and *Methanobrevibacter* (Fig. S2). This compound is known to be utilized in the methyl reaction pathway [54, 55]. As such, we speculate that it could serve as a methyl donor for CH_4_ production. This finding suggests that the role of sarcosine in enteric CH_4_ emissions from ruminants, merits further investigation. Moreover, we identified a positive correlation between proline in the rumen and sarcosine in the serum with CH_4_ parameters (Figs. S2, S3). The link between rumen proline and CH_4_ emissions has been reported in a previous study [56], suggesting that further investigation into the relationships between serum sarcosine with CH_4_ production is warranted. Overall, our results suggest a potential interaction between rumen and serum metabolites in influencing CH_4_ emissions in ruminants, which needs future investigations, especially for the role of rumen microbial metabolism in host metabolism.

## Conclusions

Although PEO did not affect animal performance or the diversity of the rumen microbiome (e.g., bacteria, archaea, and protozoa), it altered the interactions among different microbial kingdoms following its administration. Notably, the enrichment of succinate- and propionate-producing bacteria in the PEO group likely contributed to enhanced propionate metabolism in the rumen. Our results suggest that PEO administration employs diverse mechanisms of action such as formate, sulfur, methylated amines, and propionate collectively working to enhance CH_4_ inhibition while also providing alternative H_2_ sinks. Moreover, we found sarcosine in the serum metabolites, which could potentially be associated with CH_4_ reduction. It is noticeable that the functional analysis was predicted based on amplicon sequences, which has inherent limitations. Further metagenomic analysis is needed to capture the full range of microbial functions and their interactions in the rumen after PEO administration. Additionally, this study did not perform rumen samplings at different time points, which could affect the observed effects of PEO. Shaani et al. [57] noted that sampling time can influence microbial composition more than the host or diet. Therefore, future research should include multiple sampling points to fully understand the temporal dynamics of PEO’s effect on rumen microbial compositions. Regardless, our findings suggest that PEO administration could be a potential effective intervention to reduce enteric CH_4_ emissions through manipulation of rumen microbiome in goats.

## Materials and methods

### Animal ethics statement

The experimental procedures were reviewed and approved by the Gyeongsang National University Institutional Animal Care and Use Committee (protocol number: GNU-210705-E0063). The experiment took place at the Gyeongsang National University Animal Breeding Farm from November 25, 2022, to February 9, 2023.

### Experimental design, animals and diet

A total of 10 fattening Korean native goats (*Capra hircus coreanae*, 42.3 ± 1.68 kg, male) were kept in individual pens (170 × 120 cm) and were randomly divided into two dietary groups: (1) control (CON; basal diet without additive) and (2) PEO, basal diet + 1.5 g/d of PEO) using a 2 × 2 crossover design. The PEO added in this study was in liquid form and extracted from *Pinus koraiensis* pinecones, which were provided by PHYLUS (PHYLUS Co., Ltd. Seoul, Korea). The detailed information regarding the PEO extraction process and its constituents is fully described in our previous paper [22].

The orally administration dosages of PEO were determined based on our previous study [19]. To ensure the goats received the full PEO dose, we orally administered PEO aliquots in 5 mL of water using a 10 mL syringe. The CON group was given 5 mL of water. All animals were fed the same diet, consisting of tall fescue and a commercial concentrate. The chemical composition of the tall fescue and commercial concentrate are presented in Table S9. The animals were given their diet and PEO additives in two equal meals at 0800 h and 1600 h. The experimental diet included a mixture of tall fescue hay and concentrate in a 50:50 ratio, meeting the nutrient requirements based on NRC (2007) recommendations. Before providing the concentrate mix, tall fescue hay was given to encourage the goats to consume as much forage as possible. Drinking water was available at all times. The individual daily feed intake was recorded by measuring both the feed offered and any refusals. Each experimental period lasted for 28 days, with 23 days of adaptation followed by 5 days of data and sample collection. Additionally, there was a 21 days wash-out period between the two experimental periods.

### Nutritional analysis of feed

Dried feed samples (tall fescue and concentrate) were ground through a 1 mm sieve using a Wiley Mill (Arthur Thomas CO., Philadelphia, PA). The ground samples were sent to Cumberland Valley Analytical Services Inc. (Waynesboro, PA) for wet chemistry analysis. The analysis included measurements of DM, crude protein (CP), ether extract (EE), ash, minerals, amylase-treated neutral detergent fiber (aNDF), acid detergent fiber (ADF), neutral detergent insoluble crude protein (NDICP), acid detergent insoluble crude protein (ADICP), lignin, and starch. The respective analysis methods used were referenced as follows: DM (AOAC International, 2000 [58]; method 930.15), CP (AOAC International, 2000 [58]; method 990.03), EE (AOAC International, 2006 [59]; method 2003.05), ash (AOAC International, 2000 [58]; method 942.05), minerals (AOAC International, 2000 [58]; method 985.01), aNDF ([60]), ADF (AOAC International, 2000 [58]; method 973.18), NDICP and ADICP (analyzed using Leco FP-528 N Combustion Analyzer lignin [61], and starch [62]. Non-fiber carbohydrate (NFC) were calculated using Hall's equation [63]; NFC = 100 – [(CP – NDICP) + EE + ash + NDF]. The OARDC Summative Energy Equation, as described by Weiss [64], was utilized for calculating the net energy for maintenance.

### Methane measurements and data processing

Enteric CH_4_ emissions were quantified using LMD (LMm-G; Tokyo Gas Engineering Co. Ltd, Tokyo, Japan) following the procedure described by Roessler et al. [65] and Kang et al. (2022) with minor modifications. In brief, CH_4_ emissions were measured twice a day during four consecutive days (24–27 days), specifically before feed intake (0600–0800 h) and after feed intake (0900–1100 h). The CH_4_ concentration in the breathing air was continuously monitored at an interval of 0.5 s for a duration of 8 min, recorded in ppm-m. Eructation, which is the main source of CH_4_ emissions, typically occurs during the B-sequence of rumen contractions. These contractions happen irregularly, with a frequency of approximately once every 1–3 min [66]. To adequately capture CH_4_ emissions, the measurement duration was set to 8 min, as recommended by Kang et al. (2022), which allowed for the detection of 3–4 eructation events. The detailed measurement method is fully described in our previous paper [19].

The measured CH_4_ concentrations were determined using method described by Kang et al. [29], where the AMPD package in R was used to identify the peaks in the measured CH_4_ concentration data obtained from LMD. The data peaks were separated into two categories (respiration and eructation) using the mixdist R package, and each category was analyzed separately. The mean of the normal distribution was assumed to be the representative CH_4_ concentration of the gas exhaled from the track for the hour. The values of CH_4_ concentration measured four times a day were averaged to represent the mean daily CH_4_ concentration.

### Sample collection and analyses

#### Rumen fluid sampling

Before morning feeding, rumen contents were collected from each animal using oral stomach tubing (length of 150 cm and a diameter of 0.8 cm). To reduce saliva contamination, the first 20 mL of each rumen fluid sample was discarded. Subsequently, collected rumen fluid from each goat was filtered through 4 layers of cheesecloth and measured their pH with a pH meter (S220, Mettler-Toledo, Greifensee, Switzerland). After filtering the rumen fluid (10 mL), it was divided into two separate aliquots for volatile fatty acid (VFA) and ammonia nitrogen (NH_3_-N) analysis. Another 5 mL of filtered rumen fluid was centrifuged at 20,000 × *g* for 10 min at 4 °C. The supernatant was discarded, and the pellet was stored at − 80 °C for microbial analysis. All aliquots were transported to the laboratory (with dry ice) and stored at − 80 °C until further analysis.

#### Rumen fermentation parameters analyses

For VFA processing, rumen fluid samples of 1 mL were subjected to centrifugation at 20,000×*g* for 10 min at 4 °C. The resulting supernatant was utilized for analysis using a high-performance liquid chromatography system (L-2200, Hitachi, Tokyo, Japan) equipped with a UV detector (L-2400; Hitachi) and a column (MetaCarb 87H; Varian, Palo Alto, CA, USA). Concentration of the NH_3_-N was determined using a spectrophotometer (Model 680, Bio-Rad Laboratories, Hercules, CA, USA) by measuring the optical density at 630 nm. The measurement was conducted following the protocol described by Chaney and Marbach [67].

#### Blood sampling and analyses

On d 28 of each sampling period before the morning feeding, blood from the jugular neck vein was collected in a serum-separating tube (BD Vacutainer, SSTTM II advance, Becton Dickinson Co., Franklin Lakes, NJ, USA) from goats. The blood samples were centrifuged for 15 min at 1006×*g* at 4 °C, and the serum was stored at − 80 °C until analysis. The content of serum ALT/SGPT, AST/SGOT, BUN, inorganic phosphate, and glucose were measured using the UV spectrophotometry method by a Cobas 8000 c702 analyzer (Roche Diagnostics, Mannheim, Germany). The contents of albumin, total cholesterol, creatinine, total protein, triglyceride, and calcium were measured using the colorimetry method by a Cobas 8000 c702 analyzer (Roche Diagnostics, Mannheim, Germany).

#### Fecal sampling and analysis

To estimate DM digestibility, fecal samples were collected thrice on a 12-h basis during each experimental period. Within 6 h of excretion, each fecal sample was weighed and placed in individual bag. The fecal samples were dried in a forced-air oven at 55 °C for 96 h until their weight stabilized. The detailed information regarding the fecal sampling process is fully described in our previous paper [19].

#### Multi-kingdom amplicon library preparation and sequencing analyses

Total DNA of rumen fluid (1.8 mL) was extracted using the repeat bead-beating plus column method [68]. The quality and quantity of extracted DNA were evaluated using a NanoDrop spectrophotometer (ND-2000, Thermo Fisher Scientific Inc., Waltham, MA, USA). Rumen microbiota was analyzed using metataxonomic based on kingdom-specific phylogenetic markers (16S rRNA gene for bacteria and archaea [69] and 18S rRNA gene for protozoa [70]. Extracted gDNA was submitted to Macrogen (Seoul, Korea) for library preparation each primer regions (Table S10). Preparation of the amplicons barcoded library was based on the Illumina 16S rRNA and 18S rRNA amplicon sequencing library preparation protocol and the sequencing was performed using Illumina MiSeq platSform (San Diego, CA, USA). The obtained amplicon sequencing data were analyzed using Quantitative Insights Into Microbial Ecology 2 (QIIME2, v. 2021.11; [71]).

Briefly, adapter and primer sequences of bacteria and protozoa were removed using Cutadapt [72] followed by quality filtering (Q-score > 20), denoising, merging, and chimeric sequence removal as done previously using q2-dada2’s denoise-pair method [73]. Amplicon sequence variants (ASVs) were clustered at 99% similarity. Taxonomic classifiers for each kingdom were manually trained using the Naïve Bayes classifier [74] with the Silva (SSU138) gene database for bacteria and archaea. To classify protozoal ASVs, a BLASTn search was performed against the NCBI nucleotide collections (excluding uncultured/environmental samples accessed on May 3, 2023). The taxonomy of each ASV was then determined by selecting the best BLASTn hit. Major phyla and genera each representing ≥ 0.1% of total sequences in at least one of the dietary treatments were discussed in this report.

#### Analysis of sequencing data

Analysis of the differential relative abundances of rumen prokaryotes and protozoa was evaluated using Microbiome Multivariable Associations with Linear Models (MaAsLin2) package in R [75]. Differential abundance was calculated using Centered Log-Ratio normalization and LM method, with experimental period and treatment as fixed effects and individual animal as random effects.

We excluded ASVs identified as unassigned, mitochondria, and chloroplast before downstream analysis. To reduce the sampling heterogeneity, the ASV table was rarefied to the same reads per sample (ASVs) with 1000 times using the ‘q2-repeat-rarefy’ plugin from QIIME2 [76]. Microbial diversity was evaluated within samples (alpha diversity) or between samples (beta diversity) on rarefied ASVs table. Alpha diversity was evaluated using richness (Chao1 estimates), Evenness, Simpson’s index, and Shannon’s index. Beta diversity was evaluated using phylogenetic distance of Bray Curtis and Weighted UniFrac. Prediction of the metabolic functions of the rumen microbial communities was performed using PICRUSt2 (v.2.4.1) [77] and CowPI [24] to predict the functional profile of the microbial communities based on the 16S rRNA gene sequences obtained. Since the web server for CowPI is unavailable, the tool was reconstructed using precalculated files deposited on Zenodo (https://zenodo.org/record/1252858). Since MaAsLin2 did not detect predicted functional feature differences in the CowPI and PICRUSt2, linear discriminant analysis effect size (LEfSe) analysis was employed to find differences [78].

To understand relationship among the bacteria, archaea, and protozoa (relative abundance ≥ 0.1%) in the CON and PEO groups, the co-occurrence network analysis was generated using the 'FastSpar’ [79] which use SparCC algorithm [80]. The raw amplicon sequences from this study were deposited in the NCBI Sequence Read Archive (accession numbers: PRJNA975721).

#### Metabolomic analysis using proton nuclear magnetic resonance (^1^H-NMR)

Rumen fluid samples for the metabolite analysis were centrifuged it at 12,902×*g* for 10 min at 4 °C, resulting in the collection of 300 μL of the supernatant. Subsequently, 300 μL of a standard buffer solution containing (2,2,3,3-d(4)-3-(trimethylsilyl) propionic acid [TSP] sodium salt) in deuterium oxide (D_2_O) solvent/standard buffer solution (300 μL) was added to the supernatant. The combined supernatants (600 μL) were transferred to 5 mm NMR tubes for ^1^H-NMR spectroscopy analysis. This sample pre-treatment was following the procedures described by Saleem et al. [81]. Similarly, serum samples for the metabolite analysis were centrifuged at 14,000×*g* for 10 min at 4 °C. The supernatant (200 μL) was added to 400 μL of saline buffer (NaCl 0.9% w/v in 100% D_2_O) in 5 mm NMR tube for ^1^H-NMR spectroscopy analysis [82]. The spectra of rumen fluid and serum were acquired using SPE-800 MHz NMR-MS spectrometer (Bruker BioSpin AG, Fällanden, Switzerland) equipped with a 5 mm triple-resonance inverse cryoprobe featuring Z-gradients (Bruker BioSpin Co., Billerica, Massachusetts, USA). The Carr-Purcell-Meiboom-Gill pulse sequence was employed for acquiring the NMR spectra of rumen fluid and serum. Data acquisition involved collecting 64,000 data points with 128 transients, using a spectral width of 16,025.641 Hz, a relaxation delay of 4.0 s, and an acquisition time of 2.0 s [82].

#### Metabolomics data processing and analyses

The analyzed spectral data was utilized for metabolites identification and quantification using the Chenomx NMR suite 8.4 software (ChenomxInc, Edmonton, Alberta, Canada). The classification process involved the utilization of three metabolite databases: Bovine Metabolome Database (www.bovinedb.ca), the Livestock Metabolome Database (www.lmdb.ca), and the Human Metabolome Database (www.hmdb.ca). Statistical analyses of the metabolite data were performed using MetaboAnalyst 5.0 (http://www.metaboanalyst.ca). The obtained data were processed using normalization-selected methods, involving sample normalization with constant sum, data transformation through log normalization, and data scaling through auto scaling. The rumen fluid and serum metabolite data with 50% of samples under the identification limit or with at least 50% of values missing were eliminated from the analysis. Metabolic pathway analysis utilized the *Bos taurus* pathway library from the Kyoto Encyclopedia of Genes and Genomes (KEGG) website (http://www.kegg.com).

The “randomForest (RF)” package in R was used for the RF analysis [83]. The rumen metabolites were used as inputs in the RF model. For each metabolite, a mean decrease accuracy score was calculated based on the increase in error caused by removing that metabolite from the predictors. This score reflects the importance of metabolites in the model. The best predictive model was identified based on the maximum AUC, using the “pROC package” in R [84]. To minimize potential overfitting, we applied a tenfold cross-validation approach using the "trainControl" package in R [85].

To predict the probabilities of co-occurrence between microbial genera and metabolites in host rumen fluid, we employed mmvec neural network-based approach, which infers the nature of interactions across omics datasets [25]. The interactions between microbes and metabolites were ranked and visualized through the standard dimensionality reduction interface that is implemented as a plugin in QIIME2 (Version 2021.2).

### Statistical analysis

Statistical analyses were performed using SAS (v. 9.4, SAS Institute Inc., Cary, NC) and R software (v. 4.0.2). The normality of data was investigated with a Shapiro–Wilk test prior to all statistical analyses. The data obtained from in vivo experiment was analyzed using PROC GLIMMIX procedure according to the following statistical model:

$${\text{Y}}_{{{\text{ijk}}}} = \mu + {\text{A}}_{{\text{i}}} + {\text{P}}_{{\text{j}}} + \, + {\text{T}}_{{\text{k}}} + \left( {{\text{PT}}} \right)_{{{\text{jk}}}} + \varepsilon_{{{\text{ijk}}}}$$

where, Y_ijk_ = observed dependent variable, μ = overall mean, A_i_ = random effect of animal, P_j_ = fixed effect of period, T_k_ = fixed effect of treatment, (PT)_jk_ = fixed effect of interaction between period and treatment, and ε_ijk_ = unexplained error. The Wilcoxon rank-sum test was utilized for data with an abnormal distribution, and the *P* values were subsequently adjusted using FDR correction. The resulting distance matrices served as inputs for principal coordinates analysis (PCoA) and significance of sample clustering was analyzed by permutational multivariate analysis of variance (PERMANOVA) with 9,999 permutations. Regarding MaAsLin2, Benjamini–Hochberg FDR [86] adjusted *Q*-values < 0.1 were considered as significant. LEfSe uses a nonparametric factorial Kruskal Wallis and Wilcoxon rank sum test followed by a linear discriminate analysis to estimate the effect size of each taxon [78]. A significance level of *P* < 0.05 and effect size threshold of 2 were applied in the trial to identify the biomarker functional features. Comparison of each exclusive networks were accomplished with Co-expression Differential Network Analysis (CoDiNA) [23]. To define network statistics, we used the built-in plugins in Gephi (v. 0.9.2) [87] to calculate measurements of centrality (e.g., eigenvector centrality and authority). The univariate Student’s t-test was used to quantify differences between in the metabolite profiles of the rumen fluid and serum under the CON and PEO groups. Afterward, metabolite profiles resulting from the analysis of NMR data were used as explanatory variables in a partial least squares discriminant analysis (PLS-DA) to investigate how they contributed to difference after PEO administration. The variable importance in projection method (VIP) was applied to assess the relevance of each metabolite considering the ordinary VIP ≥ 1.5 threshold, in order to identify the most important features. *P* values were corrected for FDR [86] and *P* < 0.05 and 0.05 ≤ *P* < 0.01 were considered as significant and tendency effects, respectively.

### Supplementary Information

Additional file 1

## Data Availability

All data presented in this study are available from the corresponding author on reasonable request.

## Abbreviations

ALT/SGPT: Alanine transaminase/serum glutamic pyruvate transaminase
AST/SGOT: Aspartate aminotransferase/serum glutamic oxaloacetic transaminase
BW: Body weight
CH_4_: Methane
CON: Control
DDMI: Digestible dry matter intake
DM: Dry matter
DMD: Dry matter digestibility
DMI: Dry matter intake
FDR: False discovery rate
H_2_: Hydrogen
NFC: Non-fiber carbohydrate
NH_3_-N: Ammonia nitrogen
PCoA: Principal coordinates analysis
PERMANOVA: Permutational multivariate analysis of variance
RF: Random forest
VFA: Volatile fatty acid
VIP: Variable importance in projection
