# Supplementary material for: Metataxonomic and metabolomic profiling revealed Pinus koraiensis cone essential oil reduced methane emission through affecting ruminal microbial interactions and host-microbial metabolism
Source: Anim Microbiome. 2024 Jun 28;6:37. doi: 10.1186/s42523-024-00325-4 (PMC11212255; doi:10.1186/s42523-024-00325-4)
Supplement: Supplementary file 1 — Additional file 1 [file 42523_2024_325_MOESM1_ESM.docx]

**Table S1.** Comparison of rumen bacterial phylum^1^ between CON and PEO in goats

| Item | Treatments | | SEM | *P*-value^2^ | FDR^3^ |
| --- | --- | --- | --- | --- | --- |
|  | CON | PEO |  |  |  |
| Bacteroidota | 51.0 | 50.6 | 2.59 | 0.876 | 1.000 |
| Verrucomicrobiota | 21.4 | 13.3 | 3.24 | 0.037 | 0.267 |
| Firmicutes | 14.9 | 16.2 | 1.52 | 0.403 | 0.687 |
| Proteobacteria | 5.18 | 11.0 | 2.05 | 0.273 | 0.585 |
| Fibrobacterota | 2.39 | 4.75 | 1.06 | 0.089 | 0.267 |
| Spirochaetota | 1.64 | 1.25 | 0.29 | 0.089 | 0.267 |
| Cyanobacteria | 1.41 | 0.84 | 0.32 | 0.087 | 0.267 |
| Desulfobacterota | 0.49 | 0.40 | 0.13 | 0.427 | 0.687 |
| Synergistota | 0.46 | 0.45 | 0.25 | 0.966 | 1.000 |
| Thermoplasmatota | 0.29 | 0.16 | 0.04 | 0.014 | 0.210 |
| Patescibacteria | 0.28 | 0.33 | 0.12 | 1.000 | 1.000 |
| Unclassified Bacteria | 0.26 | 0.25 | 0.07 | 0.967 | 1.000 |
| Euryarchaeota | 0.05 | 0.15 | 0.04 | 0.121 | 0.303 |
| Elusimicrobiota | 0.04 | 0.11 | 0.06 | 0.675 | 0.920 |
| Others | 0.18 | 0.17 | 0.02 | 0.458 | 0.687 |

SEM: standard error of mean; FDR: false discovery rates; CON: without PEO; PEO: *Pinus koraiensis* cone essential oil.

^1^Relative abundance of major bacteria phylum (relative abundance ≥ 0.1% in more than 50% animals) for all individuals.

^2^ *P*-value obtained from non-parametric Wilcoxon rank-sum test.

^3^False discovery rate-adjusted *P*-value.

**Table S2.** Comparison of rumen bacterial families^1^ between CON and PEO in goats

| Item | Treatments | | SEM | *P*-value^2^ | FDR^3^ |
| --- | --- | --- | --- | --- | --- |
|  | CON | PEO |  |  |  |
| *Prevotellaceae* | 23.4 | 30.3 | 2.18 | 0.014 | 0.078 |
| WCHB1-41 | 18.4 | 11.3 | 2.73 | 0.032 | 0.113 |
| *Rikenellaceae* | 7.95 | 7.38 | 0.97 | 0.559 | 0.752 |
| *Bacteroidales* RF16 group | 7.92 | 5.38 | 1.33 | 0.093 | 0.227 |
| *Bacteroidales* UCG-001 | 5.02 | 2.08 | 1.04 | 0.012 | 0.078 |
| *Succinivibrionaceae* | 4.30 | 9.95 | 2.32 | 0.212 | 0.396 |
| F082 | 3.90 | 2.96 | 0.49 | 0.092 | 0.227 |
| *Clostridia* vadinBB60 group | 3.22 | 2.28 | 0.55 | 0.129 | 0.280 |
| *Erysipelatoclostridiaceae* | 3.07 | 2.43 | 0.45 | 0.195 | 0.396 |
| *Fibrobacteraceae* | 2.39 | 4.75 | 1.06 | 0.089 | 0.227 |
| *Lachnospiraceae* | 1.73 | 2.65 | 0.36 | 0.023 | 0.090 |
| *Acholeplasmataceae* | 1.71 | 1.98 | 0.59 | 0.649 | 0.755 |
| vadinBE97 | 1.65 | 0.86 | 0.24 | 0.012 | 0.078 |
| *Spirochaetaceae* | 1.60 | 1.24 | 0.30 | 0.076 | 0.227 |
| *Gastranaerophilales* | 1.41 | 0.84 | 0.32 | 0.087 | 0.227 |
| UCG-010 | 1.20 | 1.15 | 0.37 | 0.889 | 0.945 |
| *Bacteroidales* p-251-o5 | 1.07 | 0.30 | 0.29 | 0.017 | 0.078 |
| *Acidaminococcaceae* | 0.94 | 0.92 | 0.17 | 0.921 | 0.945 |
| *Victivallaceae* | 0.93 | 0.89 | 0.49 | 0.520 | 0.724 |
| *Selenomonadaceae* | 0.87 | 2.02 | 0.39 | 0.006 | 0.078 |
| Unclassified *Rhodospirillales* | 0.84 | 1.00 | 0.47 | 0.678 | 0.755 |
| *Bacteroidales* BS11 gut group | 0.63 | 0.75 | 0.34 | 0.621 | 0.755 |
| *Oscillospiraceae* | 0.60 | 0.40 | 0.07 | 0.018 | 0.078 |
| *Synergistaceae* | 0.46 | 0.45 | 0.25 | 0.273 | 0.466 |
| *Desulfovibrionaceae* | 0.45 | 0.37 | 0.14 | 0.427 | 0.641 |
| *Muribaculaceae* | 0.34 | 1.18 | 0.16 | 0.301 | 0.489 |
| RF39 | 0.31 | 0.63 | 0.27 | 0.345 | 0.538 |
| *Methanomethylophilaceae* | 0.29 | 0.16 | 0.04 | 0.014 | 0.078 |
| *Oligosphaeraceae* | 0.25 | 0.18 | 0.06 | 0.275 | 0.466 |
| Clostridia UCG-014 | 0.23 | 0.21 | 0.09 | 0.623 | 0.755 |
| *Christensenellaceae* | 0.21 | 0.66 | 0.08 | 0.000 | 0.000 |
| *Hungateiclostridiaceae* | 0.21 | 0.20 | 0.07 | 0.678 | 0.755 |
| *Saccharimonadaceae* | 0.17 | 0.20 | 0.07 | 0.970 | 0.970 |
| *Monoglobaceae* | 0.16 | 0.11 | 0.07 | 0.517 | 0.724 |
| *Ruminococcaceae* | 0.14 | 0.23 | 0.02 | 0.006 | 0.078 |
| *Marinilabiliaceae* | 0.13 | 0.11 | 0.06 | 0.607 | 0.755 |
| *Absconditabacteriales* (SR1) | 0.11 | 0.13 | 0.05 | 0.909 | 0.945 |
| *Methanobacteriaceae* | 0.05 | 0.15 | 0.04 | 0.121 | 0.278 |
| Others | 1.75 | 1.29 | 0.36 | 0.213 | 0.396 |

SEM: standard error of mean; FDR: false discovery rates; CON: without PEO; PEO: *Pinus koraiensis* cone essential oil.

^1^Relative abundance of major bacteria families (relative abundance ≥ 0.1% in more than 50% animals) for all individuals.

^2^ *P*-value obtained from non-parametric Wilcoxon rank-sum test.

^3^False discovery rate-adjusted *P*-value.

**Table S3.** Comparison of rumen bacterial genera^1^ between CON and PEO in goats

| Item | Treatments | | SEM | *P*-value^2^ | FDR^3^ |
| --- | --- | --- | --- | --- | --- |
|  | CON | PEO |  |  |  |
| WCHB1-41 | 18.4 | 11.3 | 2.73 | 0.032 | 0.210 |
| *Prevotella* | 15.3 | 21.6 | 3.66 | 0.212 | 0.463 |
| *Bacteroidales* RF16 group | 7.92 | 5.38 | 1.33 | 0.093 | 0.295 |
| *Rikenellaceae* RC9 gut group | 7.53 | 7.20 | 0.99 | 0.739 | 0.872 |
| *Bacteroidales* UCG-001 | 5.02 | 2.08 | 1.04 | 0.012 | 0.143 |
| *Erysipelatoclostridiaceae* UCG-004 | 3.07 | 2.41 | 0.44 | 0.179 | 0.422 |
| F082 | 2.96 | 3.90 | 0.49 | 0.092 | 0.295 |
| *Prevotellaceae* UCG-001 | 2.85 | 3.76 | 1.56 | 0.521 | 0.715 |
| *Fibrobacter* | 2.38 | 4.75 | 1.06 | 0.089 | 0.295 |
| *Clostridia* vadinBB60 group | 2.28 | 3.22 | 0.55 | 0.129 | 0.362 |
| *Prevotella 9* | 1.85 | 1.06 | 1.50 | 0.850 | 0.946 |
| vadinBE97 | 1.65 | 0.86 | 0.24 | 0.012 | 0.143 |
| *Succinivibrio* | 1.63 | 3.30 | 1.04 | 0.427 | 0.681 |
| UG *Prevotellaceae* | 1.61 | 1.48 | 0.59 | 0.678 | 0.818 |
| *Anaeroplasma* | 1.59 | 1.84 | 0.59 | 0.679 | 0.818 |
| UG *Succinivibrionaceae* | 1.57 | 0.45 | 0.69 | 0.595 | 0.782 |
| *Gastranaerophilales* | 1.41 | 0.84 | 0.32 | 0.087 | 0.295 |
| *Prevotellaceae* UCG-003 | 1.34 | 0.90 | 0.22 | 0.080 | 0.295 |
| UCG-010 | 1.20 | 1.15 | 0.37 | 0.889 | 0.953 |
| *Treponema* | 1.07 | 0.91 | 0.28 | 0.104 | 0.307 |
| p-251-o5 | 1.07 | 0.30 | 0.29 | 0.017 | 0.143 |
| *Succiniclasticum* | 0.94 | 0.92 | 0.17 | 0.921 | 0.953 |
| *Victivallaceae* | 0.93 | 0.89 | 0.49 | 0.520 | 0.715 |
| *Bacteroidales* BS11 gut group | 0.63 | 0.75 | 0.34 | 0.621 | 0.782 |
| *Succinivibrionaceae* UCG-002 | 0.55 | 3.95 | 1.77 | 0.241 | 0.490 |
| *Ruminobacter* | 0.51 | 2.14 | 0.89 | 0.273 | 0.520 |
| *Lachnospiraceae* ND3007 group | 0.49 | 0.40 | 0.11 | 0.467 | 0.695 |
| *Desulfovibrio* | 0.45 | 0.37 | 0.14 | 0.427 | 0.681 |
| *Fretibacterium* | 0.39 | 0.40 | 0.26 | 0.341 | 0.582 |
| *Muribaculaceae* | 0.34 | 1.18 | 0.76 | 0.162 | 0.416 |
| RF39 | 0.31 | 0.63 | 0.27 | 0.345 | 0.582 |
| *Rikenellaceae* SP3-e08 | 0.31 | 0.13 | 0.13 | 0.241 | 0.490 |
| *Prevotellaceae* YAB2003 group | 0.30 | 1.19 | 0.50 | 0.089 | 0.295 |
| *Oscillospiraceae* UCG-002 | 0.29 | 0.11 | 0.05 | 0.095 | 0.295 |
| *Sphaerochaeta* | 0.27 | 0.20 | 0.04 | 0.088 | 0.295 |
| *Butyrivibrio* | 0.25 | 0.36 | 0.04 | 0.038 | 0.224 |
| UG *Lachnospiraceae* | 0.24 | 0.38 | 0.14 | 0.970 | 0.970 |
| *Clostridia* UCG-014 | 0.23 | 0.21 | 0.09 | 0.623 | 0.782 |
| *Selenomonas* | 0.22 | 0.42 | 0.08 | 0.007 | 0.143 |
| *Lachnospiraceae* XPB1014 group | 0.21 | 0.16 | 0.04 | 0.262 | 0.515 |
| *Christensenellaceae* R-7 group | 0.21 | 0.66 | 0.08 | 0.000 | 0.000 |
| *Quinella* | 0.20 | 0.74 | 0.42 | 0.909 | 0.953 |
| *Veillonellaceae* UCG-001 | 0.20 | 0.17 | 0.03 | 0.315 | 0.563 |
| UG *Methanomethylophilaceae* | 0.20 | 0.10 | 0.03 | 0.017 | 0.143 |
| *Oligosphaeraceae* horsej-a03 | 0.18 | 0.14 | 0.05 | 0.471 | 0.695 |
| *Anaerovibrio* | 0.18 | 0.47 | 0.06 | 0.021 | 0.155 |
| *Candidatus Saccharimonas* | 0.17 | 0.20 | 0.07 | 0.970 | 0.970 |
| *Saccharofermentans* | 0.17 | 0.16 | 0.05 | 0.850 | 0.946 |
| *Monoglobus* | 0.16 | 0.11 | 0.07 | 0.517 | 0.715 |
| *Lachnospiraceae* AC2044 group | 0.16 | 0.29 | 0.08 | 0.170 | 0.418 |
| UG *Marinilabiliaceae* | 0.13 | 0.11 | 0.06 | 0.607 | 0.782 |
| *Oscillospiraceae* NK4A214 group | 0.12 | 0.15 | 0.02 | 0.144 | 0.386 |
| *Acholeplasmataceae* NED5E9 | 0.12 | 0.10 | 0.05 | 0.448 | 0.695 |
| *Absconditabacteriales* (SR1) | 0.11 | 0.13 | 0.05 | 0.909 | 0.953 |
| *Lachnoclostridium* | 0.11 | 0.16 | 0.08 | 0.307 | 0.563 |
| *Alloprevotella* | 0.09 | 0.15 | 0.05 | 0.089 | 0.295 |
| *Ruminococcus* | 0.07 | 0.12 | 0.02 | 0.014 | 0.143 |
| *Methanobrevibacter* | 0.05 | 0.14 | 0.04 | 0.206 | 0.463 |
| Others | 3.99 | 4.31 | 0.59 | 0.850 | 0.946 |

SEM: standard error of mean; FDR: false discovery rates; CON: without PEO; PEO: *Pinus koraiensis* cone essential oil.

^1^Relative abundance of major bacteria genera (relative abundance ≥ 0.1% in more than 50% animals) for all individuals.

^2^ *P*-value obtained from non-parametric Wilcoxon rank-sum test.

^3^False discovery rate-adjusted *P*-value.

**Table S4.** Exclusive network statistics of rumen microbiota between CON and PEO.

| Item | Treatments | |
| --- | --- | --- |
|  | CON | PEO |
| Nodes | 22 | 27 |
| Total edges | 21 | 48 |
| Positive | 15 | 36 |
| Negative | 6 | 12 |
| Positive (%) | 71.4 | 75.0 |
| Negative (%) | 28.6 | 25.0 |
| Abundance of exclusive node (%) | 50.0 | 59.3 |
| Network diameter | 5 | 10 |
| Graph density | 0.091 | 0.137 |
| Modularity | 0.585 | 0.367 |
| No. of communities | 5 | 5 |
| Average clustering coefficient | 0.183 | 0.255 |
| Best centrality node | *Lachnospiraceae AC2044 group* | *Anaerovibrio* |

CON: without PEO; PEO: *Pinus koraiensis* cone essential oil.

**Table S5.** Differential enrichment of metabolites content of rumen between CON and PEO in goats

| Metabolites | Classification | CON/PEO | *P*-value | FDR^1^ | VIP^2^ | FC^3^ |
| --- | --- | --- | --- | --- | --- | --- |
| Isopropanol | Alcohol | CON | 0.008 | 0.304 | 2.13 | 0.51 |
| N-Nitrosodimethylamine | Amines | CON | 0.014 | 0.324 | 2.00 | 2.49 |
| Trimethylamine | Amines | PEO | 0.077 | 0.410 | 1.52 | -1.18 |
| Proline | Amino acids | CON | 0.003 | 0.304 | 2.35 | 1.65 |
| 3,4-Dihydroxybenzeneacetate | Benzoic acids | CON | 0.044 | 0.410 | 1.71 | 2.49 |
| o-Cresol | Benzoic acids | CON | 0.064 | 0.410 | 1.58 | 0.73 |
| Syringate | Benzoic acids | PEO | 0.038 | 0.410 | 1.75 | -2.49 |
| Erythritol | Carbohydrates | PEO | 0.012 | 0.304 | 2.05 | -2.21 |
| Fructose | Carbohydrates | CON | 0.044 | 0.410 | 1.70 | 1.11 |
| Galactitol | Carbohydrates | CON | 0.065 | 0.410 | 1.58 | 1.12 |
| Glucuronate | Carbohydrates | CON | 0.062 | 0.410 | 1.60 | 1.30 |
| Lactose | Carbohydrates | PEO | 0.031 | 0.410 | 1.80 | -1.48 |
| Lactulose | Carbohydrates | PEO | 0.008 | 0.304 | 2.13 | -1.46 |
| Pyruvate | Carbohydrates | CON | 0.061 | 0.410 | 1.60 | 0.43 |
| Succinate | Carbohydrates | CON | 0.076 | 0.410 | 1.53 | 1.00 |
| 2-Hydroxyisobutyrate | Carboxylic acids | CON | 0.042 | 0.410 | 1.72 | 0.42 |
| 2-Phenylpropionate | Carboxylic acids | CON | 0.050 | 0.410 | 1.67 | 1.37 |
| Guanidoacetate | Carboxylic acids | CON | 0.089 | 0.446 | 1.47 | 1.29 |
| N-Acetylaspartate | Carboxylic acids | CON | 0.073 | 0.410 | 1.54 | 0.66 |
| N-Acetylcysteine | Carboxylic acids | PEO | 0.033 | 0.410 | 1.79 | -1.40 |
| N-Acetylglycine | Carboxylic acids | CON | 0.009 | 0.304 | 2.10 | 1.27 |
| trans-Aconitate | Carboxylic acids | CON | 0.038 | 0.410 | 1.75 | 1.30 |
| Creatinine | Imidazolinones | CON | 0.083 | 0.427 | 1.50 | 2.92 |
| 2-Hydroxyisocaproate | Lipids | CON | 0.073 | 0.410 | 1.54 | 1.44 |
| Choline | Lipids | PEO | 0.042 | 0.410 | 1.72 | -1.00 |
| Pimelate | Lipids | CON | 0.059 | 0.410 | 1.61 | 1.12 |
| Hypoxanthine | Nucleosides | CON | 0.043 | 0.410 | 1.71 | 2.00 |
| Formate | Organic acids | PEO | 0.076 | 0.410 | 1.52 | -0.84 |
| Fumarate | Organic acids | CON | 0.011 | 0.304 | 2.06 | 1.31 |
| Isobutyrate | Organic acids | CON | 0.077 | 0.410 | 1.52 | 0.57 |
| Malate | Organic acids | CON | 0.010 | 0.304 | 2.09 | 1.99 |
| O-Acetylcholine | Organic acids | CON | 0.036 | 0.410 | 1.76 | 1.03 |
| Propionate | Organic acids | PEO | 0.031 | 0.410 | 1.81 | -0.29 |
| 3-Phenylpropionate | Others | PEO | 0.054 | 0.410 | 1.64 | -0.43 |
| Cellobiose | Others | CON | 0.054 | 0.410 | 1.64 | 1.14 |
| Dimethyl sulfone | Others | PEO | 0.067 | 0.410 | 1.57 | -1.28 |

^1^False discovery rate-adjusted *P*-value.

^2^Variable importance in the projection obtained from partial least square-discriminant analysis model.

^3^Fold change; calculated as binary logarithm average abundance response ratio between CON and PEO, where the positive value means that average abundance response of the metabolites in the former is larger than that in the latter and vice versa.

CON: without PEO; PEO: *Pinus koraiensis* cone essential oil; CON/PEO, comparison between CON and PEO.

**Table S6.** Differential enrichment of metabolites content of serum between CON and PEO in goats

| Metabolites | Classification | CON/PEO | *P*-value | FDR^1^ | VIP^2^ | FC^3^ |
| --- | --- | --- | --- | --- | --- | --- |
| Kynurenine | Amines | PEO | 0.049 | 0.840 | 1.99 | -1.22 |
| Sarcosine | Amines | CON | 0.052 | 0.840 | 1.97 | 0.82 |
| Taurine | Amines | CON | 0.068 | 0.876 | 1.86 | 2.39 |
| Alanine | Amino acids | CON | 0.101 | 0.876 | 1.69 | 0.23 |
| Phenylalanine | Amino acids | CON | 0.050 | 0.840 | 1.98 | 1.88 |
| Acetylsalicylate | Benzoic acids | CON | 0.023 | 0.840 | 2.25 | 1.77 |
| Syringate | Benzoic acids | CON | 0.063 | 0.876 | 1.89 | 1.12 |
| 2-Phenylpropionate | Carboxylic acids | CON | 0.096 | 0.876 | 1.71 | 0.99 |
| N-alpha-Acetyllysine | Carboxylic acids | PEO | 0.076 | 0.876 | 1.82 | -1.92 |
| N-Phenylacetylphenylalanine | Carboxylic acids | CON | 0.038 | 0.840 | 2.08 | 1.38 |
| Allantoin | Imidazolinones | CON | 0.099 | 0.876 | 1.70 | 1.46 |
| 3-Indoxylsulfate | Indoles | CON | 0.044 | 0.840 | 2.03 | 1.42 |
| 2-Hydroxyvalerate | Lipids | CON | 0.046 | 0.840 | 2.01 | 1.65 |
| O-Acetylcarnitine | Lipids | PEO | 0.027 | 0.840 | 2.20 | -2.21 |
| Thymol | Lipids | PEO | 0.086 | 0.876 | 1.76 | -0.68 |
| Acetoin | Others | CON | 0.049 | 0.840 | 1.99 | 2.35 |
| Galactarate | Others | CON | 0.080 | 0.876 | 1.80 | 0.95 |
| Theophylline | Others | CON | 0.034 | 0.840 | 2.12 | 1.00 |

^1^False discovery rate-adjusted *P*-value.

^2^Variable importance in the projection obtained from partial least square-discriminant analysis model.

^3^Fold change; calculated as binary logarithm average abundance response ratio between CON and PEO, where the positive value means that average abundance response of the metabolites in the former is larger than that in the latter and vice versa.

CON: without PEO; PEO: *Pinus koraiensis* cone essential oil; CON/PEO, comparison between CON and PEO.

**Table S7.** Pathway analysis of significantly different rumen metabolites compared with CON and PEO in goats

| Metabolic pathways | Total Cmpd | Hits^1^ | *P*-value | -Log (*P*-value) | FDR^2^ | Impact^3^ |
| --- | --- | --- | --- | --- | --- | --- |
| Alanine, aspartate and glutamate metabolism | 28 | 4 | 0.004 | 2.404 | 0.011 | 0.09 |
| Amino sugar and nucleotide sugar metabolism | 37 | 1 | 0.029 | 1.534 | 0.045 | 0.00 |
| Aminoacyl-tRNA biosynthesis | 48 | 1 | 0.003 | 2.539 | 0.010 | 0.00 |
| Arginine and proline metabolism | 38 | 3 | 0.001 | 2.954 | 0.007 | 0.10 |
| Arginine biosynthesis | 14 | 1 | 0.007 | 2.160 | 0.017 | 0.00 |
| Ascorbate and aldarate metabolism | 10 | 1 | 0.036 | 1.446 | 0.045 | 0.25 |
| Citrate cycle (TCA cycle) | 20 | 4 | 0.002 | 2.782 | 0.008 | 0.15 |
| Galactose metabolism | 27 | 1 | 0.048 | 1.318 | 0.056 | 0.00 |
| Glycerophospholipid metabolism | 36 | 2 | 0.012 | 1.939 | 0.024 | 0.03 |
| Glycine, serine and threonine metabolism | 34 | 3 | 0.012 | 1.915 | 0.024 | 0.02 |
| Glyoxylate and dicarboxylate metabolism | 32 | 3 | 0.003 | 2.561 | 0.010 | 0.00 |
| Inositol phosphate metabolism | 30 | 1 | 0.036 | 1.446 | 0.045 | 0.00 |
| Pentose and glucuronate interconversions | 18 | 1 | 0.036 | 1.446 | 0.045 | 0.13 |
| Propionate metabolism | 23 | 2 | 0.020 | 1.698 | 0.036 | 0.00 |
| Purine metabolism | 66 | 1 | 0.030 | 1.525 | 0.045 | 0.02 |
| Pyruvate metabolism | 22 | 3 | 0.001 | 3.287 | 0.007 | 0.24 |
| Tyrosine metabolism | 42 | 3 | 0.001 | 2.975 | 0.007 | 0.03 |

Total Cmpd, The total number of compounds in the pathway

^1^The actually matched number from the user uploaded data.

^2^False discovery rate-adjusted P-value.

^3^The pathway impact value calculated from pathway topology analysis.

**Table S8.** Pathway analysis of significantly different serum metabolites compared with CON and PEO in goats

| Metabolic pathways | Total Cmpd | Hits^1^ | *P*-value | -Log (*P*-value) | FDR^2^ | Impact^3^ |
| --- | --- | --- | --- | --- | --- | --- |
| Alanine, aspartate and glutamate metabolism | 28 | 1 | 0.010 | 2.009 | 0.043 | 0.00 |
| Selenocompound metabolism | 20 | 1 | 0.010 | 2.009 | 0.043 | 0.00 |
| Tryptophan metabolism | 41 | 1 | 0.014 | 1.841 | 0.043 | 0.09 |
| Aminoacyl-tRNA biosynthesis | 48 | 2 | 0.026 | 1.585 | 0.059 | 0.00 |

Total Cmpd, The total number of compounds in the pathway

^1^The actually matched number from the user uploaded data.

^2^False discovery rate-adjusted P-value.

^3^The pathway impact value calculated from pathway topology analysis.

**Table S9.** Mineral and chemical composition of experimental diets fed to goats.

| Item^1^ | Tall fescue | Concentrate^2^ |
| --- | --- | --- |
| Mineral composition (% of DM) | | |
| Ash | 8.21 | 9.46 |
| Calcium | 0.25 | 1.25 |
| Phosphorus | 0.19 | 0.66 |
| Magnesium | 0.18 | 0.32 |
| Potassium | 2.36 | 1.24 |
| Sodium | 0.04 | 0.36 |
| Iron, mg/kg | 159 | 366 |
| Manganese, mg/kg | 40.0 | 128 |
| Zinc, mg/kg | 20.0 | 174 |
| Copper, mg/kg | 6.00 | 34.0 |
| Chemical composition (% of DM) | | |
| Dry matter | 92.9 | 89.5 |
| Moisture | 7.10 | 10.5 |
| Crude protein | 11.6 | 16.4 |
| Soluble protein | 4.50 | 6.22 |
| Ether extract | 1.52 | 4.14 |
| Starch | 1.23 | - |
| NDF | 48.6 | 33.7 |
| NDICP | 1.20 | 4.37 |
| ADF | 26.8 | 16.6 |
| ADICP | 0.66 | 1.25 |
| Lignin | 2.18 | 4.50 |
| Non-fiber carbohydrates^2^ | 31.3 | 36.3 |
| Energy, Mcal/lb^3^ | | |
| Metabolizable energy | 0.67 | 1.17 |
| Net energy maintenance | 0.71 | 0.76 |
| Net energy gain | 0.44 | 0.49 |
| Total digestible nutrients (% of DM) | 65.2 | 68.2 |

SEM: standard error of the mean; DM: dry matter. NDF: neutral detergent fiber; NDICP: neutral detergent insoluble crude protein; ADF: Acid detergent fiber; ADICP: acid detergent insoluble crude protein.

^1^Chemical analysis was performed by Cumberland Valley Analytical Services (Waynesboro, PA, USA).

^2^The formulation of the concentrate used in this study contained crude protein (13.5% or more), ether extract (2.5% or more), crude fiber (20.0% or less), ash (10.0% or less), calcium (0.80% or more), phosphate (0.80% or less), and TDN 67% (Values are concentrations declared by the manufacturer).

^3^According to Hall (2000) equation.

^4^Net energy for maintenance was calculated using the OARDC Summative Energy Equation of Weiss (1998).

**Table S10.** Primers (F = forward, R = reverse) for real-time PCR assay

| Target species | Primer sequence (5′→3′) | Reference |
| --- | --- | --- |
| 16S rRNA gene | 515F: GTG YCA GCM GCC GCG GTA A | [71] |
|  | 806R: GGA CTA CNV GGG TWT CTA AT |  |
| 18S rRNA gene | RP841F: GAC TAG GGA TTG GAG TGG | [72] |
|  | Reg1320R: AAT TGC AAA GAT CTA TCC C |  |

^a^bp, base pair. F: forward; R: reverse.

Figures


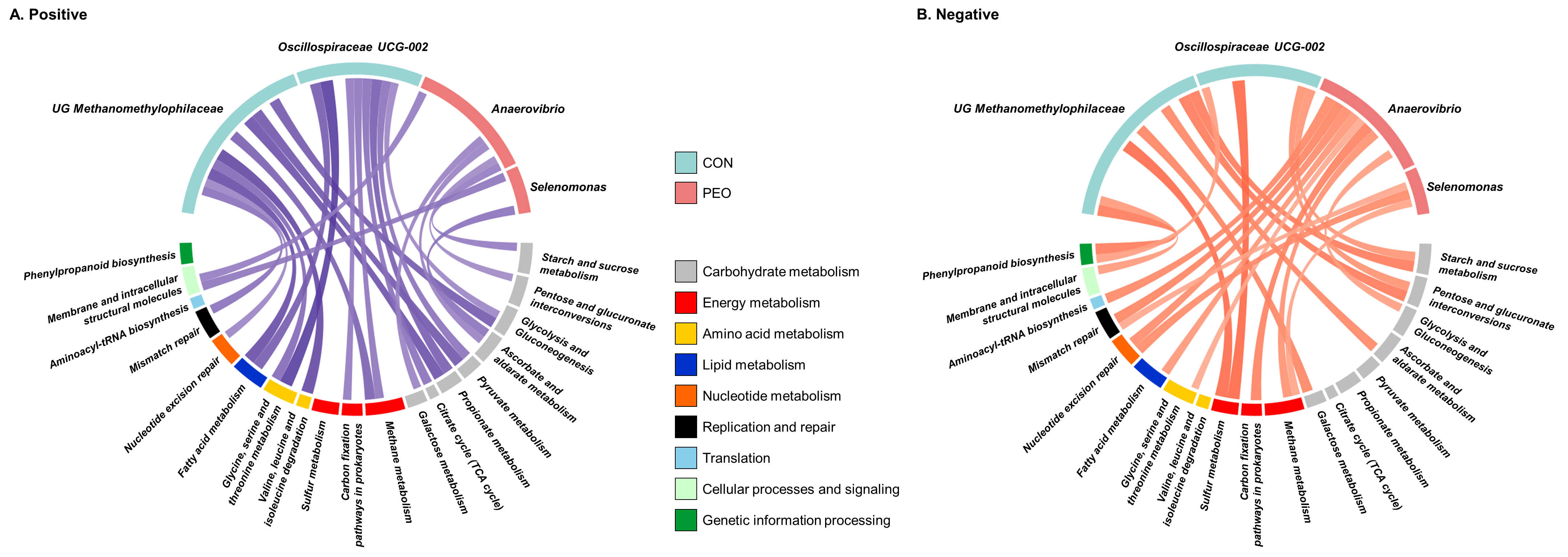


**Figure S1.** Spearman’s rank correlations between rumen microbiota and predicted functional features. Only strong (correlation coefficient |*r*| ≥ 0.6) and significant (*P* < 0.05) correlations were selected to be displayed. Color intensity represent the magnitude correlation. **A** positive correlation (blue); **B** negative correlation (red). Functional feature was predicted using CowPI database.


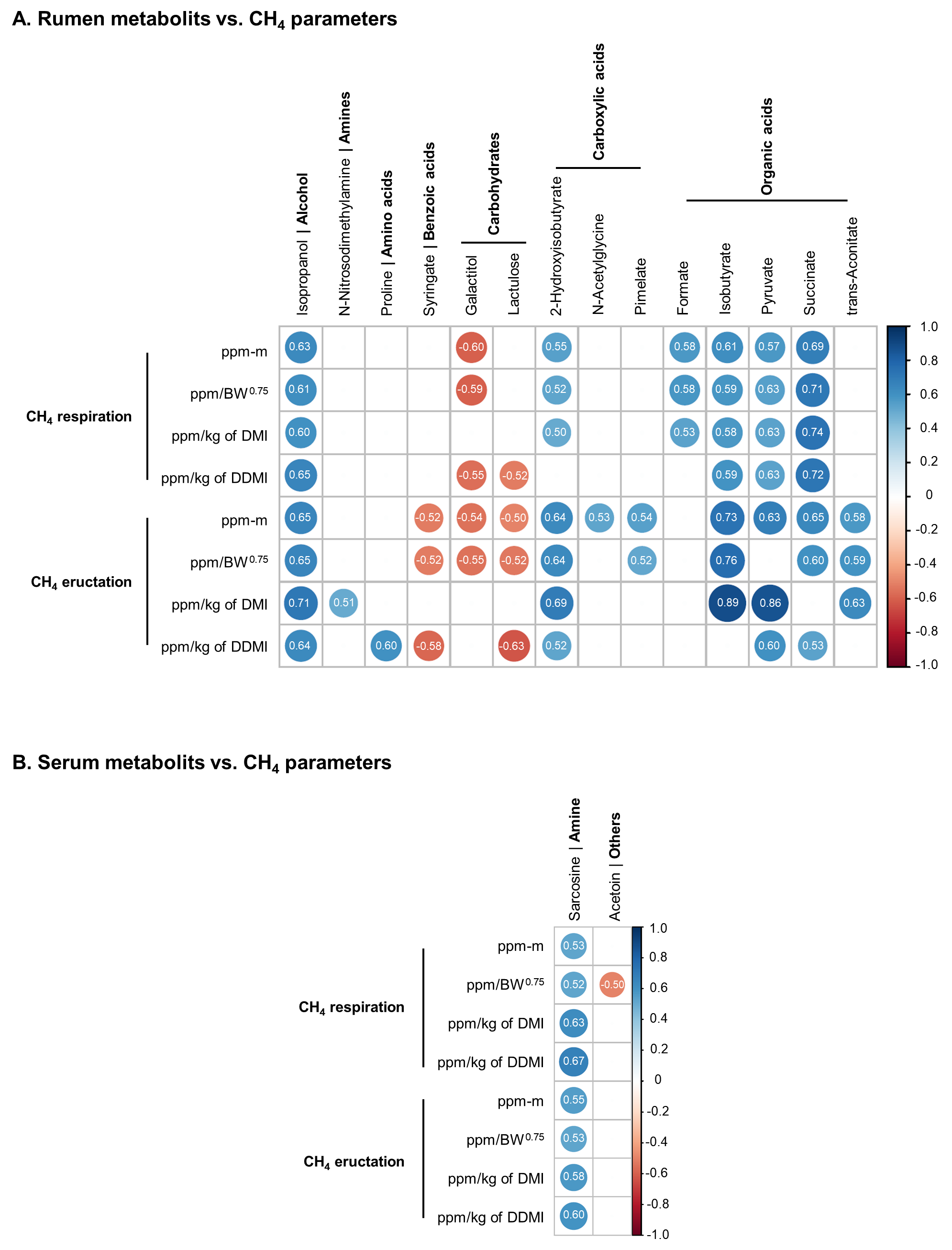


**Figure S2.** Correlation of **A** rumen and **B** serum metabolites with the methane emission parameters. Correlation analyses were conducted using Spearman’s rank correlation. Only strong correlation coefficients (|*r*| ≥ 0.5) and significant (*P* < 0.05) correlations were selected to be shown on the plot. CH_4_, methane.


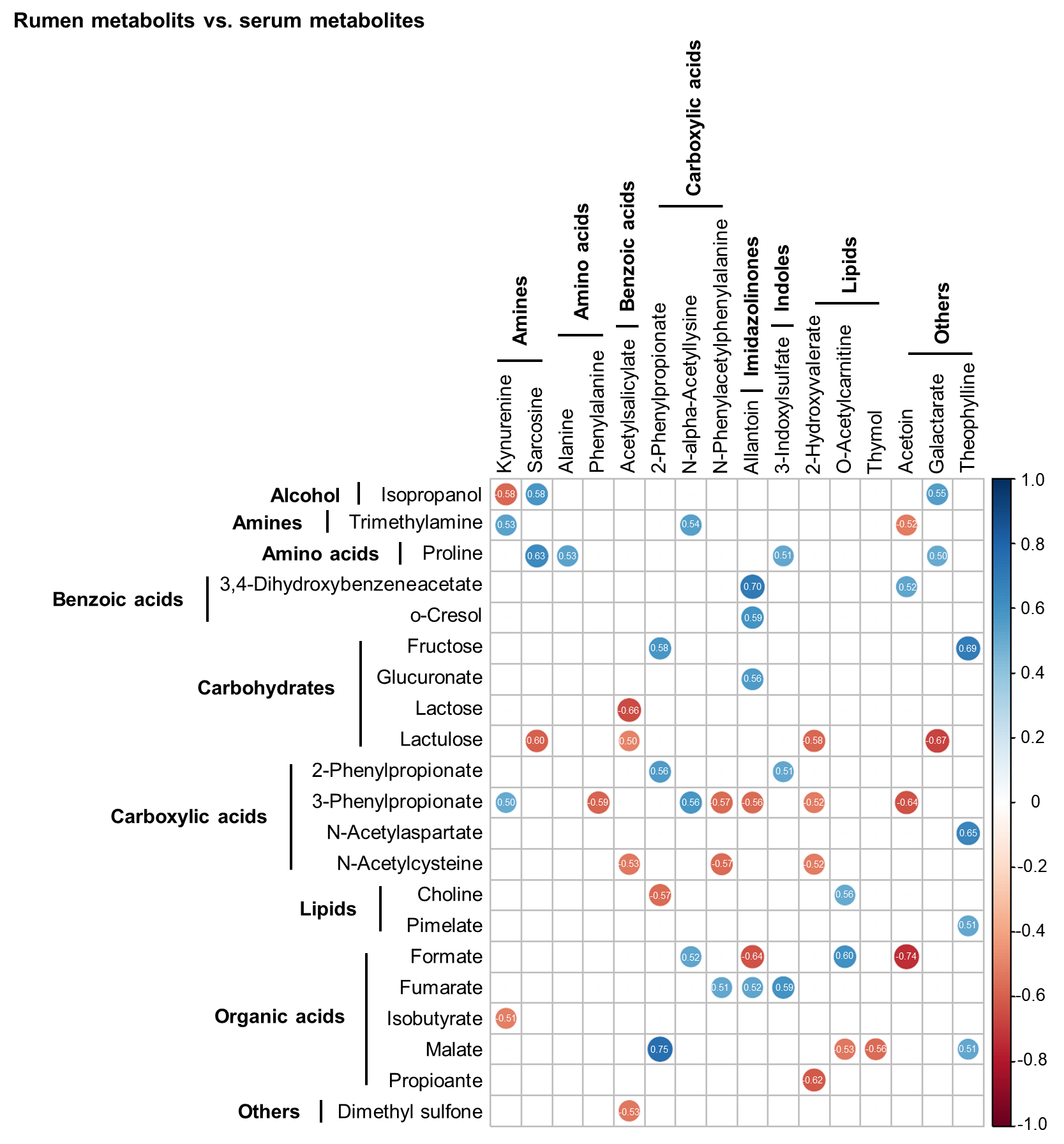


**Figure S3.** Correlation of rumen and serum metabolites. Correlation analyses were conducted using Spearman’s rank correlation. Only strong correlation coefficients (|*r*| ≥ 0.5) and significant (*P* < 0.05) correlations were selected to be shown on the plot. The horizontal axis represents rumen fluid, while the vertical axis represents serum.
